# Strain- and chirality-engineered tunability of electronic and thermoelectric properties in SiC nanotubes: insights from first-principles calculations

**DOI:** 10.1039/d6ra00892e

**Published:** 2026-04-14

**Authors:** Imam Hussain, A. S. M. Jannatul Islam, Tamanna Tanvin Tanha, Md. Mafizul Islam

**Affiliations:** a Department of Electrical and Electronic Engineering, Khulna University of Engineering & Technology Khulna 9203 Bangladesh jannatul@eee.kuet.ac.bd; b Department of Textile Machinery Design and Maintenance, Bangladesh University of Textiles Dhaka 1208 Bangladesh

## Abstract

Silicon carbide nanotubes (SiCNTs) have recently emerged as promising candidates for next-generation thermoelectric applications, owing to their moderate bandgaps and intrinsically lower lattice thermal conductivity—attributable to strong quantum confinement effects. In realistic situations, uniaxial strain arising from lattice mismatch, thermal gradients, or dynamic motion in wearables can significantly modulate the electronic and thermoelectric performance of these nanostructures. In this work, we systematically investigate the strain-dependent electronic and thermoelectric properties of four representative single-walled SiCNTs—three zigzag [(6,0), (10,0), and (11,0)] and one armchair [(6,6)]—using first-principles calculations coupled with Boltzmann transport theory. The results reveal distinct chirality-dependent responses to both compressive and tensile strains. The (6,6) armchair SiCNT exhibits an indirect bandgap of 2.63 eV, while all zigzag SiCNTs show direct bandgaps under strain, with (6,0) showing the narrowest gap (0.49 eV). The Seebeck coefficient remains stable (∼1550 µV K^−1^) for the (6,6), (10,0), and (11,0) tubes across strain regimes, suggesting robustness suitable for strain-insensitive thermoelectric devices. Conversely, the (6,0) tube displays a broad Seebeck tunability (821–1550 µV K^−1^), offering potential for adaptive or strain-sensing applications. Strain engineering substantially enhances the thermoelectric power factor (PF/*τ*), achieving values of 1.36 × 10^14^ mW m^−1^ K^−2^ s^−1^ and 2.07 × 10^14^ mW m^−1^ K^−2^ s^−1^ for (10,0) and (11,0) tubes, respectively, at −2% and −10% strain, and 1.33 × 10^14^ mW m^−1^ K^−2^ s^−1^ for the (6,0) tube under maximum compressive strain. The (6,6) tube shows a ∼1.17 × PF/*τ* enhancement under compressive loading. Strain modulates normalized electrical conductivity, increasing from ∼1.29 × 10^19^ (Ω ms)^−1^ to 2.25 × 10^19^ (Ω ms)^−1^ in the (6,6) tube under −10% strain, while tensile strain reduces conductivity in (6,0), (10,0) and (11,0) tubes. Normalized electronic thermal conductivity also exhibits chirality-dependent strain sensitivity, ranging from 9.20 × 10^13^ W m^−1^ K^−1^ s^−1^ in (6,0) to >14.69 × 10^13^ W m^−1^ K^−1^ s^−1^ in (6,6) under −10% strain. Notably, the thermoelectric figure of merit (*ZT*) of the pristine (6,0) SiCNT at 300 K attains a value of ∼0.27, highlighting its promising thermoelectric efficiency relative to carbon nanotubes. These findings provide valuable design insights for the development of flexible, high-performance SiCNT-based nanodevices, including strain sensors, energy harvesters, and thermoelectric coolers.

## Abstract 2

Strain and chirality synergistically tune SiCNT electronic structure and thermoelectric performance enabling high efficiency flexible nanoenergy devices.

## Graphical abstract

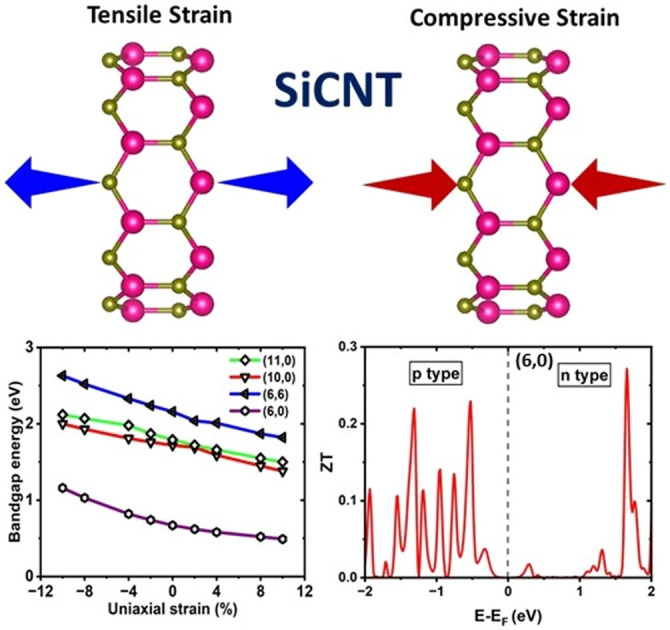

## 1. Introduction

The global demand for wearable electronic devices—such as wireless sensor networks, fiber transistors, fabric antennae, flexible interconnects, fiber-based circuitry, smartwatches, and health monitoring systems—has been rapidly increasing.^1,2^ These technologies critically depend on power sources that are flexible, lightweight, compact, and capable of delivering continuous and reliable energy.^3^ However, the rapid depletion of fossil-based non-renewable resources, coupled with the escalating energy demands of industrial processes, underscores the urgent need for sustainable, environmentally benign alternatives.^4,5^ Remarkably, a vast fraction of energy generated worldwide is dissipated as heat, regardless of whether it originates from fossil fuels or renewable resources. Harnessing this otherwise wasted heat and directly converting it into electricity represents a transformative route for powering mobile and wearable electronics.^6^ Besides, the human body itself represents an abundant and underutilized energy reservoir, where physiological heat dissipation and biomechanical activities—such as breathing, walking, or even finger movements—can serve as sustainable energy inputs for wearable devices.^7,8^

Motivated by this potential, significant research has been devoted to solid-state nanogenerators that harvest mechanical or thermal energy based on piezoelectric, triboelectric, electrostatic, and thermoelectric principles.^9–11^ Among these, solid-state thermoelectric devices, which exploit the Seebeck effect to convert temperature gradients into electrical energy, offer a compelling solution.^12^ Unlike conventional energy harvesters, thermoelectric nanogenerators are characterized by the absence of moving parts, zero greenhouse gas emissions, high stability, and extended operational lifetimes, making them uniquely suited for wearables and self-powered systems.^6,13,14^ Beyond power generation, these devices are also capable of active thermal management through the Peltier effect, enabling localized cooling and heating functionalities. Consequently, thermoelectric nanogenerators have found increasing applications in self-powered biosensors and IoT-enabled healthcare devices.^7,14,15^ Generally, the thermoelectric performance of a material is commonly quantified by the dimensionless figure of merit (*ZT*), defined as *ZT* = *S*^2^*σTκ*^−1^, where *σ* is the electrical conductivity, *S* is the Seebeck coefficient, *κ* is the thermal conductivity, and *T* is the absolute temperature. For efficient cooling or heating, a high *κ* is beneficial, whereas for energy harvesting applications, maximizing the power factor (PF = *S*^*2*^*σ*) while minimizing *κ* is essential.^16^

Commercial inorganic thermoelectric materials such as Bi_2_Te_3_, PbTe, SnSe, and their alloys are well known for their high Seebeck coefficients and excellent thermoelectric performance near room temperature.^17–19^ However, their intrinsic drawbacks—including limited abundance, high cost, and mechanical rigidity—severely constrain their use in wearable technologies. In contrast, organic thermoelectric materials, despite exhibiting relatively low power factors, offer compelling advantages such as non-toxicity, mechanical flexibility, and cost-effectiveness.^20^ Semiconducting polymers, for instance, typically deliver modest *ZT* values (0.1–0.42) under ambient conditions, attributed to their inherently low thermal conductivity (∼1.5 W m^−1^ K^−1^), though their power factors (78–500 µW m^−1^ K^−2^) remain substantially lower than that of state-of-the-art inorganic counterparts.^21,22^ To address these limitations, hybrid strategies have emerged. Inorganic thermoelectric materials are often coated onto flexible substrates^23^ or embedded into elastomeric polymers with compliant terminals,^24^ enabling mechanical adaptability while maintaining functionality. While such approaches enhance bendability, issues such as poor stretchability, compressibility, and limited conformability to irregular body surfaces remain unsolved. This technological gap highlights an urgent need to redesign and engineer next-generation thermoelectric materials that simultaneously achieve high electrical performance and mechanical adaptability.

In this regard, low-dimensional nanostructures, particularly one-dimensional nanotubes, have emerged as promising thermoelectric materials owing to their unique electronic and thermal transport properties arising from quantum confinement of charge carriers. Presently, carbon nanotubes (CNTs), the rolled form of graphene, exhibit remarkable attributes such as mechanical flexibility and ease of fabrication; these features are highly desirable for wearable TE devices. However, the inherently high thermal conductivity of CNTs limits their thermoelectric efficiency, necessitating performance tuning through structural modifications.^25,26^ Recent advances have therefore shifted attention to silicon carbide nanotubes (SiCNTs), which combine the advantageous features of CNTs with additional merits, such as superior oxidation resistance, wide bandgaps, high melting temperature, and enhanced chemical reactivity due to electronegativity differences between Si and C atoms.^25–27^ These characteristics not only impart high structural stability but also render SiCNTs particularly suitable for biocompatible applications.^26^ Since the first successful synthesis of SiCNTs by Sun *et al.* in 2002,^28^ followed by the Taguchi group's high-temperature template-assisted synthesis of multi-walled and single-walled SiCNTs,^29,30^ various experimental strategies have been demonstrated, including chemical vapor deposition, carbothermal reduction, and template-based growth.^28–31^

Recently, numerous first-principles density functional theory (DFT) studies, alongside machine learning-assisted investigations, have focused on SiCNT systems.^32–35^ These works have systematically explored the structural, electronic, magnetic, spin-polarization, Curie temperature, and density of states (DOS) properties of single-walled SiCNTs. In particular, doping strategies have been employed to tune the properties of SiCNTs, revealing significant deviations from their pristine structures. Structurally, SiCNTs resemble CNTs, with their electronic properties dictated by chirality defined by the chiral vector *C*_h_ = *na*_1_ + *ma*_2_ = (*n*, *m*). Depending on chirality, SiCNTs can exhibit metallic, direct semiconducting, or indirect semiconducting behavior, offering versatile opportunities for electronic and thermoelectric device engineering.^8^ In addition, in nanotube-based devices, the presence of uniaxial strain is inevitable. Mechanical deformation can arise from lattice mismatch during film growth, uniaxial tension in aligned fibers, residual strain from textile processing (weaving, twisting, or knitting), thermal expansion mismatch in composites, and dynamic strains induced by motion in wearable applications.^36,37^ In nanotubes of different chirality, such strain can significantly alter the orbital hybridization, redistribute the DOS near the Fermi level, and lift or reshape band degeneracies, thereby modulating the carrier effective mass, mobility, Seebeck coefficient, power factor, and others thermoelectric properties.^38–41^ Despite numerous DFT studies on SiCNTs, a comprehensive first-principles investigation of the electronic and thermoelectric properties that simultaneously examines multiple chiralities under a wide range of strains is still missing. A detailed understanding of chirality and strain engineering in SiCNTs is very important for bridging fundamental electronic structure modulation with practical thermoelectric performance optimization.

The present work employs first principles DFT combined with the Boltzmann transport equation to comprehensively investigate the electronic and thermoelectric properties of single-walled SiCNTs under varying strains and chiralities. Three zigzag SiCNTs— (6,0), (10,0), and (11,0)—and one armchair SiCNT, (6,6) are considered in this study. The changes in band structure (E–k), DOS, Seebeck coefficient (*S*), electrical conductivity (*σ*), and power factor (PF) with uniaxial strains ranging from −10% to +10% are calculated. Among the four SiCNT samples, the thermoelectric figure of merit (*ZT*) of the (6,0) zigzag SiCNT at zero strain and room temperature is calculated to evaluate its superiority over existing CNT systems. Our results reveal that applying uniaxial strain along the nanotube axis effectively tunes the electronic bandgap as well as the thermoelectric transport coefficients (*S*, *σ*, PF), thereby offering a pathway to optimize the performance of SiCNTs for practical applications.

## 2. Computational details

To investigate the structural and electronic properties of single-walled SiCNTs (SWSiCNTs), first-principles calculations are performed within the DFT framework using the Quantum ESPRESSO simulation package.^42^ The structural optimization and property calculations utilize a plane-wave basis set and projector-augmented wave (PAW) pseudopotentials, with the Perdew–Burke–Ernzerhoff (PBE) exchange–correlation functional within the generalized gradient approximation (GGA). It is noted that the GGA-PBE functional is known to underestimate absolute bandgap values; however, it reliably captures relative trends with respect to chirality and uniaxial strain, which are central to the present thermoelectric analysis.^43^ We have studied three types of zigzag SWSiCNTs with chiralities (10,0), (11,0), (6,0), and an armchair type (6,6) SWSiCNT, with dissimilar diameters. These zigzag SWSiCNTs can be generalized as (*n*, 0) with *n* = 3*i* + *j*, where *j* = ±1 indicates semiconducting properties, and *j* = 0 indicates metallic behavior. The initial geometries are created using a TubeGen package,^44^ considering a hexagonal honeycomb lattice structure for the SWSiCNT unit cell having a Si–C bond length of 1.79 Å.^45^

For geometry optimization and self-consistent field (SCF) calculations, a gamma-centered 1 × 1 × 5 Monkhorst–Pack k-grid is used. For non-self-consistent field (NSCF) calculations, a denser 1 × 1 × 1000 k-mesh is employed for high accuracy in DOS. We set kinetic energy and charge density cutoffs at 45 Ry and 360 Ry, respectively, using the Fermi–Dirac smearing method. A 30 Å vacuum is applied perpendicular to the tube axis to isolate SWSiCNTs and prevent interaction between neighboring tubes. The geometry optimization is conducted until forces on atoms and total energy are below 0.001 Ry Bohr^−1^ and 0.0001 Ry, respectively, using the Broyden–Fletcher–Goldfarb–Shanno (BFGS) algorithm. The cutoff energy (*vs.* total energy), *k*-point density (*vs.* bandgap), and *k*-point sampling (*vs.* thermoelectric parameters) are carefully selected based on convergence tests, with the results illustrated in Fig. S1 and S2 (SI). Electronic band structures are computed within the irreducible first Brillouin zone of the hexagonal *k*-path (Γ–*M*–*K*–Γ–*A*–*L*–*H*–*A*|*L*–*M*|*K*–*H*).^46^

Uniaxial strain along the tube axis is modeled on relaxed structures, with strain percentages are calculated using 
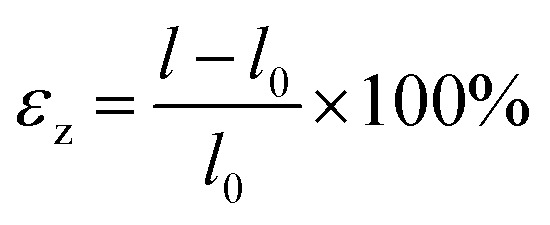
, where *l* and *l*_0_ are the strained and unstrained tube lengths, respectively. Positive values indicate tensile strain, and negative values indicate compressive strain. Strains ranging from −10% to +10% are applied *via* external hydrostatic pressures along the tube axis. At each applied axial strain, all atomic positions are fully relaxed until the residual forces are minimized. No bond breaking or structural reconstruction is observed within the considered strain range (±10%), indicating that the nanotubes remain mechanically stable within the elastic regime. Moreover, to ensure mechanical stability, the total energy *versus* the considered strain (−10% to +10%) is calculated and shown in Fig. S3 (SI). The chosen strain range shows considerably negative total energy values, ensuring structural stability, and follows earlier theoretical studies on nanotube systems aimed at exploring strain-induced electronic and transport modulation.^46^

We have estimated atomic orbitals onto the E–k plot of SWSiCNTs to understand the variation of orbitals and sub-orbitals’ contribution with strain. The electronic properties are then used to calculate thermoelectric characteristics, including the Seebeck coefficient (*S*), electrical conductivity (*σ*), thermoelectric power factor (PF), and electronic thermal conductivity (*κ*_e_). These properties are computed using semi-classical Boltzmann transport theory within the constant relaxation time approximation (CRTA) as implemented in the BoltzTraP2 code,^47^ and all transport coefficients are presented as normalized quantities with respect to the relaxation time (*τ*). The Seebeck coefficient *S*(*µ*) and electrical conductance *G*(*µ*), both as functions of chemical potential (*µ*), are given by the following expressions:1
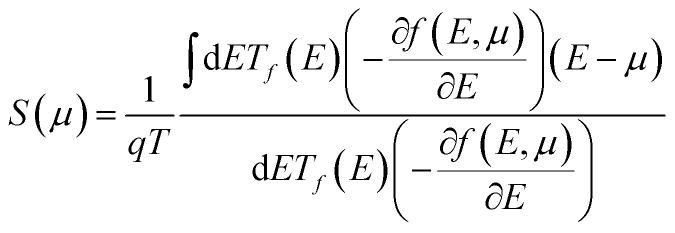
2
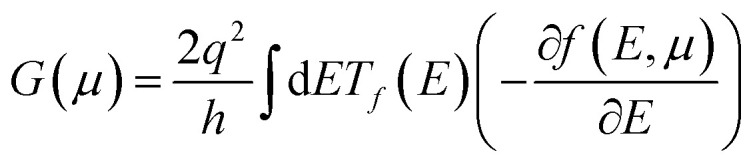
where *T* is the system temperature, *q* is the carrier charge, and *µ* is the chemical potential, which is nothing but the material's doping property. *T*_*f*_(*E*) is the transmission function shows the likelihood of carrier transit across the materials at a specific energy level. Planck's constant is represented by *h*, and the statistical distribution of electrons with a probability of occupying an energy state at a specific temperature is described by *E*, the Fermi–Dirac distribution function *f*(*E*,*µ*). At 300 K, the TE characteristics of both strained and unstrained SWSiCNTs are computed.

Finally, the thermoelectric power conversion efficiency of a system depends on its thermoelectric figure of merit (*ZT*), which can be determined using the following relation3
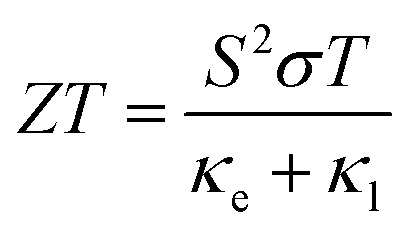
where, the Seebeck coefficient (*S*), electrical conductivity (*σ*), absolute temperature (*T*), electronic thermal conductivity (*κ*_e_) are obtained from semiclassical Boltzmann transport calculations using the BoltzTraP code, while the lattice thermal conductivity (*κ*_l_) can be obtained from the phonon transport calculations.

In this work, the lattice thermal conductivity (*κ*_*l*_) of the SWSiCNTs is calculated by solving the phonon Boltzmann transport equation (BTE) within the finite-displacement approach as implemented in the Phono3py code.^48^ Second- and third-order interatomic force constants are obtained from DFT calculations performed using the Quantum ESPRESSO package. The displaced supercells required for the anharmonic force constant calculations are generated using the Phono3py interface, and the corresponding forces are computed from SCF calculations. Among the four SiCNTs samples, considering the relatively large unit cell of the (6,0) SWSiCNT containing 24 atoms, a 1 × 1 × 1 supercell is adopted for the phonon calculations to maintain computational feasibility, as larger supercells significantly increase the number of displaced configurations required for third-order force constant evaluation.^48,49^ Due to the quasi-one-dimensional nature of the nanotube and the presence of a large vacuum region perpendicular to the tube axis, phonon sampling is performed primarily along the axial direction. Accordingly, the lattice thermal conductivity is evaluated by solving the phonon BTE on a *q*-point mesh sampled along the nanotube axis (1 × 1 × 25) at 300 K.

## 3. Result and discussion

**Fig. 1:**
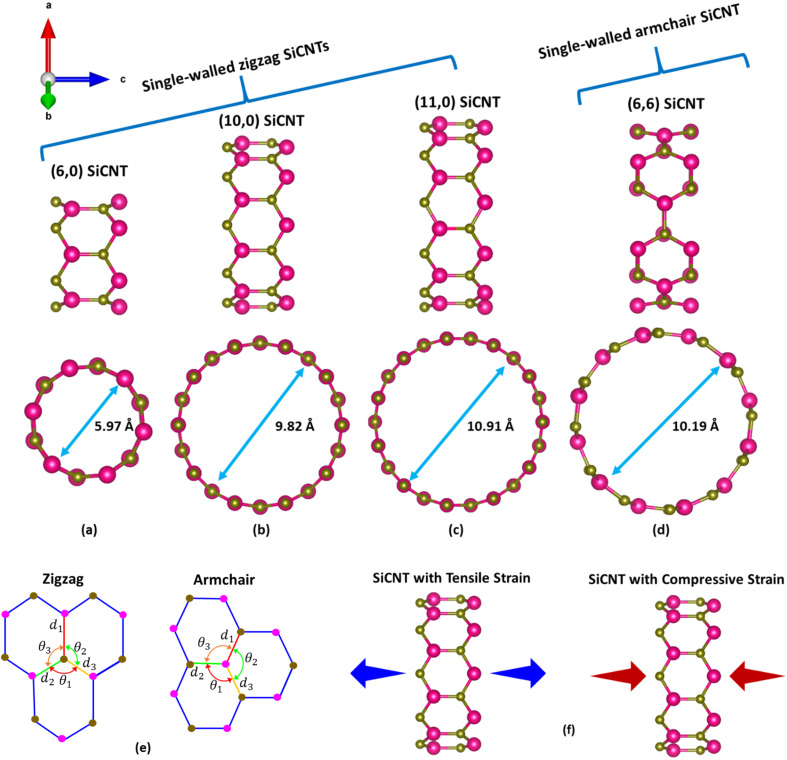
Side and top views of relaxed single-walled SiCNTs with chiral indices (a) (6,0), (b) (10,0), (c) (11,0), and (d) (6,6); (e) schematic representation of bond lengths and bond angles for zigzag and armchair configurations; (f) illustration of uniaxial tensile and compressive loading in single-walled SiCNTs.

The atomic structure of SWSiCNTs is optimized iteratively until the system reaches its minimum ground state energy. The unit cell geometries of (6,6), (6,0), (10,0), and (11,0) SWSiCNTs are initially constructed, consisting of 24, 24, 40, and 44 atoms, respectively. Moreover, the unit cell geometries of the SWSiCNTs are optimized until the total energy and atomic forces meet the predefined convergence criteria. The optimized structures of the SWSiCNTs are illustrated in Fig. 1(a)–(d). For the (6,6), (6,0), (10,0), and (11,0) SWSiCNTs, the optimized diameters are determined to be 10.19, 5.97, 9.82, and 10.91 Å, respectively. Fig. 1(e) presents a schematic representation of the bond lengths and bond angles of the optimized SWSiCNT structure. For armchair SWSiCNTs, the Si–C bond angle *θ*_1_, positioned opposite to bond length *d*_1_, is found to be slightly smaller than the corresponding angles *θ*_2_ and *θ*_3_. In zigzag SWSiCNTs, the extent of curvature-induced distortion is strongly diameter dependent. In the narrow (6,0) tube, the Si–C bond lengths remain almost the same, but the bond angles become clearly uneven: *θ*_1_ decreases to about 113°, while *θ*_2_ and *θ*_3_ increase to about 119° and 120°, showing noticeable angular distortion. This angular disparity highlights the significant strain imposed by high curvature. In contrast, the bond lengths in wider zigzag tubes such as (10,0) and (11,0) become virtually indistinguishable (∼1.7928 Å), and the bond angles converge to near-perfect uniformity (∼119.4–119.5°), closely resembling the ideal planar bonding environment of a SiC sheet. These observations underscore that increasing diameter mitigates curvature effects, progressively restoring geometric symmetry in zigzag SWSiCNTs. The bond lengths between adjacent carbon atoms and the corresponding bond angles for the relaxed SWSiCNT structures are summarized in Table 1. Our optimized geometric parameters are in good agreement with the values reported in previous studies.^50^

**Table 1:** Optimized geometrical parameters of single-walled SiCNTs, including tube diameter (*D*), silicon–carbon bond lengths (*d*), and bond angles (*θ*)

SiCNTs	*D* (Å)	*d*_1_ (Å)	*d*_2_ (Å)	*d*_3_ (Å)	*θ*_1_ (deg.)	*θ*_2_ (deg.)	*θ*_3_ (deg.)
**(6,6)**	10.19	1.78906	1.78909	1.78909	117.4703	117.4704	117.4704
**(6,0)**	5.97	1.79011	1.78991	1.78901	113.0572	119.0574	120.2827
**(10,0)**	9.82	1.79280	1.79276	1.79266	119.4426	119.4425	119.4425
**(11,0)**	10.91	1.79228	1.79228	1.79229	119.5446	119.5446	119.5450

**Fig. 2:**
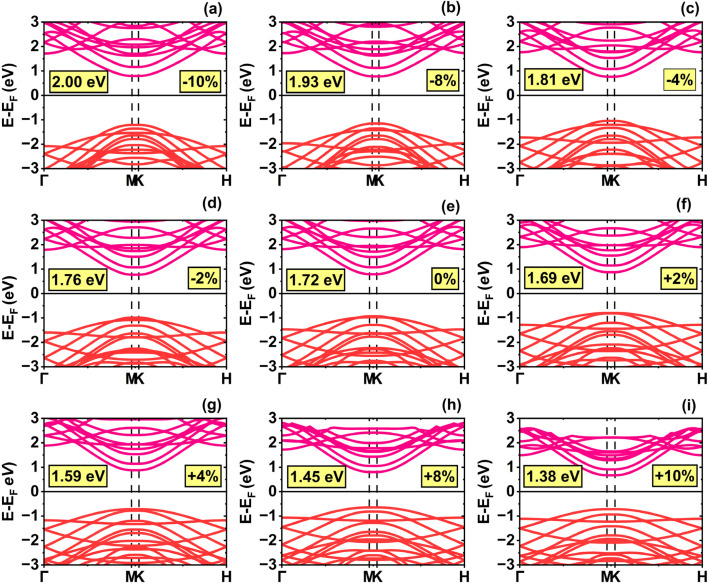
Electronic band structures of the (10,0) single-walled SiCNT under uniaxial strain: (a) −10%, (b) −8%, (c) −4%, (d) −2%, (e) 0% (relaxed state), (f) +2%, (g) +4%, (h) +8%, and (i) +10%.

**Fig. 3:**
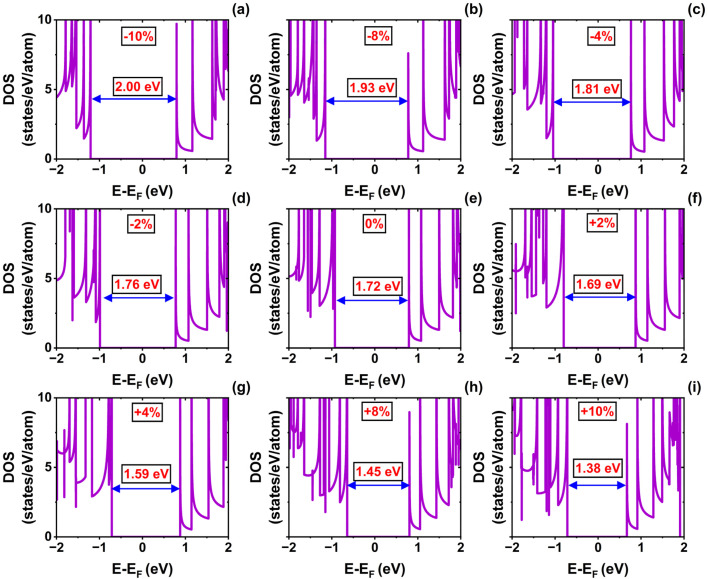
Density of states (DOS) of the (10,0) single-walled SiCNT under uniaxial strain: (a) −10%, (b) −8%, (c) −4%, (d) −2%, (e) 0% (relaxed state), (f) +2%, (g) +4%, (h) +8%, and (i) +10%.

To investigate the influence of uniaxial strain on the electronic properties of SWSiCNTs, we have calculated the band structures and DOS for four representative configurations. In this work, the calculation is performed for the first optical transition only. For the (10,0) SWSiCNT, the calculated bandgap associated with the first optical transition is 1.72 eV in the absence of strain (Fig. 2). Upon applying strain parallel to the tube axis, the bandgap is found to shrink under tensile strain, whereas an opposite behavior is observed under compressive strain in the range of −2% to −10% (see Fig. 2). Fig. 2 shows that the direct bandgap nature is preserved throughout the entire strain range, in agreement with previously reported findings for zigzag SWSiCNTs.^51^ To further elucidate our findings, the DOS for the corresponding SWSiCNTs along the tube axis is presented in Fig. 3 for comparison. As expected, tensile strain reduces the bandgap, whereas compressive strain increases it. The uniform pattern of bandgap variation under uniaxial strain can be attributed to the shift of the Dirac points (*K* and *K*′). For the (10,0) SWSiCNTs, tensile strain increases the curvature of the lowest sub-band compared to compressive strain, suggesting a lower effective mass and, in turn, enhanced charge carrier mobility under tensile strain.

For the (11,0) SWSiCNTs, a similar band structure behavior is observed (see Fig. S4). Under uniaxial strain, the (11,0) SWSiCNTs exhibit semiconducting behavior with a direct bandgap. When the SWSiCNT is stretched from its unstrained state to +10% strain, its bandgap decreases from 1.79 to 1.50 eV. As the SWSiCNTs are compressed by 10%, the bandgap rises from 1.79 to 2.12 eV. The application of compressive and tensile strain led to an increase and a decrease in the bandgap, respectively, following a trend similar to that observed in previous studies.^52^ To provide deeper insight into these results, the DOS of the corresponding SWSiCNTs along the tube axis is illustrated in Fig. S5 for comparison. Fig. S6 illustrates the effect of uniaxial strain on the electronic band structure of (6,0) SWSiCNTs. This chirality can be generally expressed as (*n*,0), where *n* is an integer multiple of 3. Due to curvature effects, the (6,0) SWSiCNT exhibits a small bandgap of 0.67 eV in the relaxed state, which is in good agreement with previously reported DFT calculations.^50^ Under compressive strain, the bandgap increases, reaching 1.16 eV at −10%. In contrast, tensile strain reduces the bandgap, decreasing to 0.62 eV at +2% and further down to 0.49 eV at +10%. This variation confirms the strong dependence of the electronic structure on axial strain. The bandgap increases under compressive strain and decreases under tensile strain, as are confirmed by the DOS calculations (see Fig. S7).

**Fig. 4:**
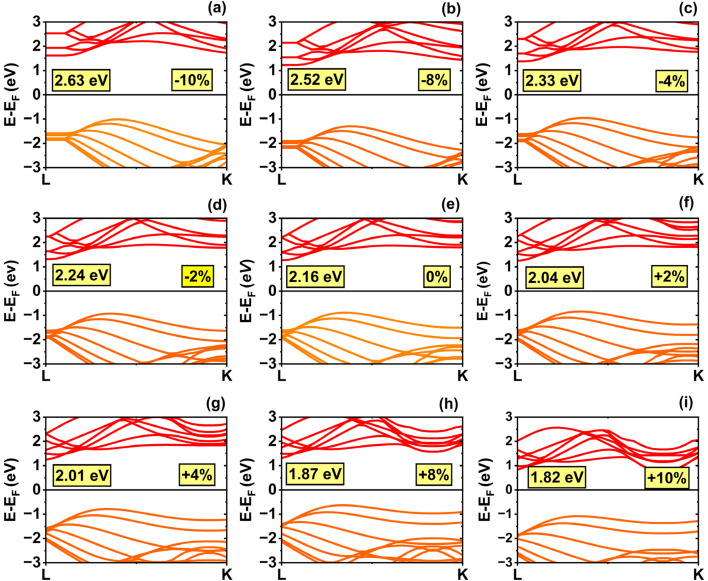
Electronic band structures of the (6,6) single-walled SiCNT under uniaxial strain: (a) −10%, (b) −8%, (c) −4%, (d) −2%, (e) 0% (relaxed state), (f) +2%, (g) +4%, (h) +8%, and (i) +10%.

**Fig. 5:**
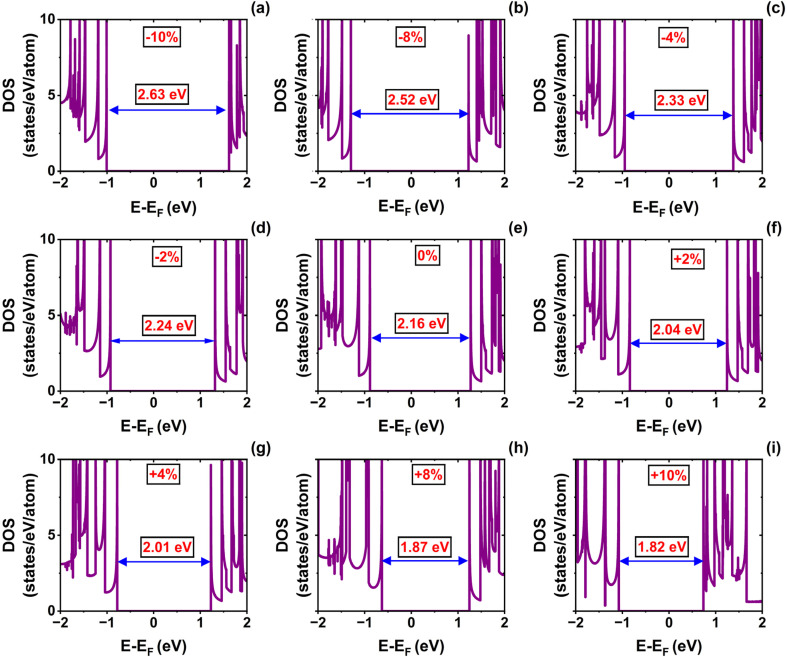
Density of states (DOS) of the (6,6) single-walled SiCNT under uniaxial strain: (a) −10%, (b) −8%, (c) −4%, (d) −2%, (e) 0% (relaxed state), (f) +2%, (g) +4%, (h) +8%, and (i) +10%.

The armchair (6,6) SWSiCNT exhibits an indirect bandgap (see Fig. 4). For these nanotubes, the bandgap increases under compressive deformation and decreases under tensile strain. At equilibrium, the (6,6) SWSiCNT exhibits an indirect bandgap of 2.16 eV, which is highly sensitive to strain. The bandgap remains relatively large at 2.24 eV under −2% strain, increases further to 2.33 eV at −4%, and reaches a maximum of 2.63 eV at −10% compressive strain. In contrast, tensile strain causes the bandgap to narrow, dropping to 2.04 eV at +2%, and further reducing it to 1.87 eV at +8%. The smallest bandgap of 1.82 eV is observed at +10% strain, thereby confirming a strong, inverse relationship between tensile strain and the bandgap. The corresponding DOS calculations confirmed the validity of the bandgap modulation (see Fig. 5).

For zigzag SWSiCNTs, the lower end of the conduction band and the upper end of the valence band are located at *M* and *K* points as the energy of the carrier becomes maximum or minimum at those points only within the *k*-path. In the case of the armchair (6,6) SWSiCNT, the desired bandgap is found along *L*–*K*. The conduction band minimum (CBM) is at the point *L*, whereas the valence band maximum (VBM) is located at about 
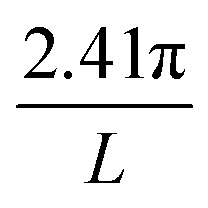
 (where *L* is the lattice constant, 3.10 Å) above the CBM. The (6,6) bandstructure shows that the VBM does not change the shape due to strain, and the VBM always remains almost at the same point found for 0% strain. But the position of CBM changes from *K* to *L* point, which creates a harmony with previous literature.^52^ The quantization of the *k* lines perpendicular to the tube axis, occurs in SWSiCNTs. In the tube axial direction, the electron wavenumber will be practically continuous, since the electrons can travel farther in the direction parallel to the SWSiCNT axis. Each quantized value's continuous states constitute a 1D sub-band, which is represented by a permitted *k* line in the 2D Brillouin zone. The discrete values of *k*_⊥_ are determined using the boundary condition along the tube circumference:

4 π*Dk*_⊥_ = 2π*t*

where *D* is the tube diameter and *t* is an integer representing the quantization index. The electronic character of SWSiCNTs depends on the alignment of allowed *k* lines with the Dirac points (*K* or *K*′): metallic behavior occurs when a *k* line crosses these points, where the π and π* bands merge, whereas a bandgap appears otherwise, resulting in semiconducting behavior. The presence of a direct bandgap suggests that zigzag SiCNTs are likely to display strong luminescence. Moreover, as the diameter of these nanotubes increases, the bandgap gradually increases in a monotonic manner until it stabilizes at a certain value. This behavior is attributed to curvature-induced σ–π hybridization, which varies with nanotube diameter—an effect also observed in boron nitride nanotubes (BNNTs).^53^ In zigzag SiCNTs, this curvature-induced σ–π mixing has a more pronounced influence compared to armchair SiCNTs, resulting in a notable decrease in the bandgap.

**Fig. 6:**
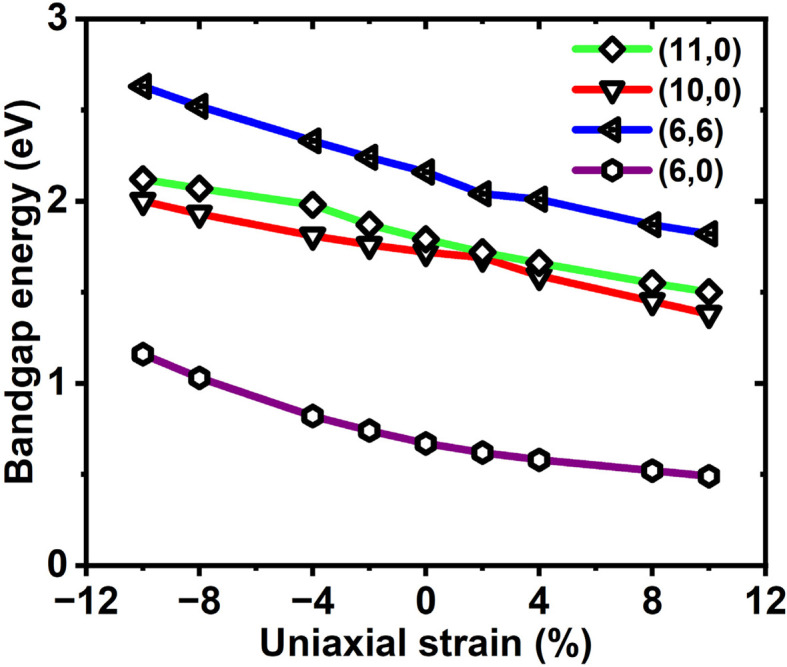
Variation of the bandgap with uniaxial strain along the tube axis for single-walled SiCNTs with (10,0), (11,0), (6,6), and (6,0) configurations.

Fig. 6 shows that, under modest strain, the bandgap varies approximately linearly with strain for both tensile and compressive deformations. For instance, within the uniaxial strain range of −10% to +10%, the bandgap exhibits nearly monotonic behavior for the zigzag (11,0), (10,0), and (6,0) SWSiCNTs, as well as for the armchair (6,6) SWSiCNT. This indicates that the bandgap can be effectively tuned over a broad energy range through uniaxial deformation. With axial tensile deformation, the bandgap of the armchair tube (6,6) and zigzag tube (10,0): (11,0): (6,0) may be adjusted between 2.16 and 1.82 eV, 1.72 and 1.38 eV: 1.79 and 1.50 eV: 0.67 eV and 0.49 eV, respectively. The (10,0), (11,0) SWSiCNTs, which lie under the (*n*,0) zigzag SWSiCNTs of type *n* = 3*i* ± 1 manifested a positive slope for the compressive strain and a negative slope for the tensile strain. All the zigzag SWSiCNT exhibits direct semiconducting behavior. In contrast, the armchair (6,6) SWSiCNT is a semiconducting nanotube with an indirect bandgap. While sharing a similar trend of bandgap variation with other zigzag SiC nanotubes, the (6,6) SWSiCNT distinguishes itself by consistently possessing the highest bandgap among them. On the other hand, it is well known that the GGA-PBE functional systematically underestimates bandgap values due to self-interaction errors and the lack of exchange–correlation derivative discontinuity. While this may affect the absolute magnitude of the bandgap, previous studies^54^ have shown that PBE generally preserves the overall band dispersion and effective mass trends, which are critical for analyzing transport behavior. To assess the reliability of our results, we have added a comparative assessment of bandgap values obtained using LDA,^55^ GGA-PBE (present calculations), GGA-PBE (reported literature values),^56^ and hybrid HSE06 estimates,^57^ as summarized in Table S1 of the SI. Although hybrid calculations predict larger bandgaps, the qualitative trends remain consistent, which is critical for evaluating strain-dependent thermoelectric transport.

**Fig. 7:**
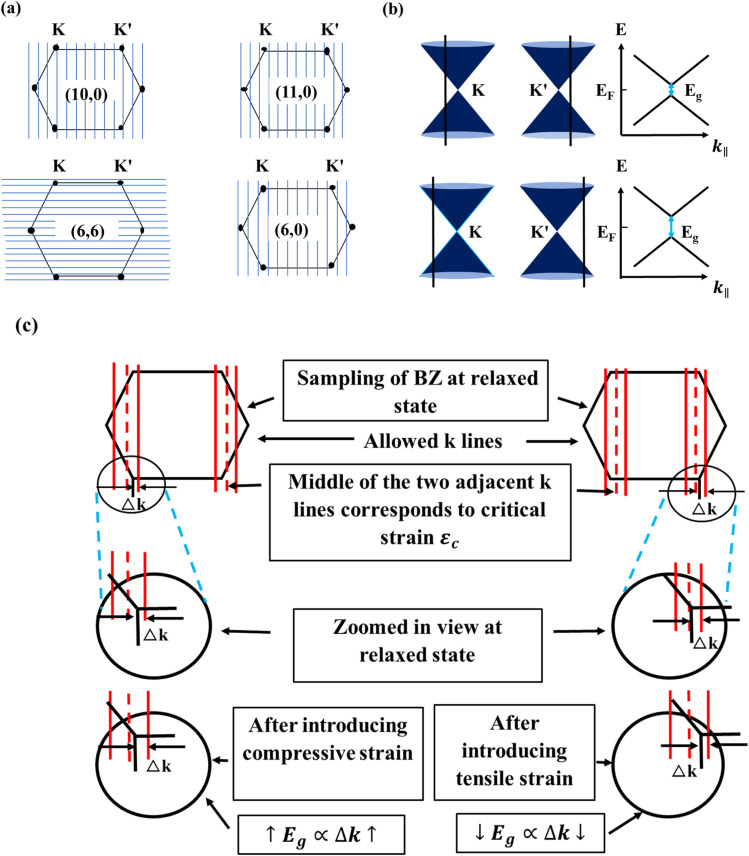
(a) Sampling of irreducible first Brillouin zones (BZ) for (10,0), (11,0), (6,6), and (6,0) single-walled SiCNTs. (b) Dirac points (*K* and *K*′) and the energy dispersion cones under uniaxial strain along the tube axis, showing the opening of a bandgap in the 1D sub-bands. The displacement of *K* and *K*′ relative to the allowed cutting lines is shown in the inset. (c) Exaggerated view of the irreducible first BZ sampling for the (10,0) SiCNT, with compressive strain on the left and tensile strain on the right.

The strain-induced bandgap variations across these nanotubes can be attributed to the modification of the first Brillouin zone's sampling, which alters the electronic band structure. The Brillouin zone of a SWSiCNT has a hexagonal shape, with Dirac points positioned at the corners—specifically at the *K* and *K*′ points—where the π and π* bands cross each other. When the *k*-line intersects the vertex of the hexagonal Brillouin zone—corresponding to the Dirac points—it indicates a zero-bandgap, signifying metallic behavior in SWSiCNTs. In contrast, for zigzag SWSiCNTs, where *n* = 3*i* + 1 or *n* = 3*i* − 1, the *k*-line does not intersect the Dirac points, resulting in semiconducting behavior characterized by a finite bandgap. Applying tensile strain to both armchair and zigzag SWSiCNTs led to a decrease in the bandgap, as the Dirac points in the Brillouin zone are initially far away from the nearest *k*-line. This phenomenon has been clearly discussed in Fig. 7(c), taking (10,0) as a sample. Under strain, the *k*-line shifted close to the Dirac points, causing the spacing Δ*k* between the nearest allowed cutting line and the Dirac point (*K* or *K*′) to reduce. Hence, the bandgap *E*_g_ begin to decrease once the Dirac points move towards a *k*-line, thereby reducing the Δ*k*, since *E*_g_ ∝ Δ*k*. But within our short range of strain variation, no crossing of the critical line occurs, which ensures no semiconductor-to-metallic conversion is valid. This shift led to a uniform-like trend in the variation of *E*_g_ under uniaxial strain. For the (10,0) and (11,0) type SiCNTs, the *E*_g_ varies linearly with strain, with a change of ∼31 meV for every 1% change of strain, whereas for (6,6) and (6,0) types it is ∼21 meV and ∼33.5 meV, respectively. The uniform variation of bandgap is observed in Fig. 6, and the reason for the opening of bandgap is illustrated in Fig. 7(b) and (c). In its pristine state, the (10,0) SiC nanotube is semiconducting as its quantized energy levels avoid the Dirac points, but compressive strain shifts these points and levels apart, consequently opening a bandgap. The trend in *E*_g_ variation observe in our analysis aligns well with previously reported experimental and simulation findings.^52^

To gain deeper insight into the origin of strain-induced bandgap modulation, orbital-resolved projected DOS (PDOS) calculations are performed. As shown in Fig. S8, the VBM is predominantly composed of C-2p states, whereas the CBM is mainly governed by Si-3p orbitals. The strain-induced variation in Si-3p–C-2p hybridization near the band edges explains the observed changes in bandgap under compressive and tensile strain. Moreover, at this stage, it is important to explicitly link the electronic structure characteristics with the transport behavior discussed later. The strain-induced redistribution of the DOS near the band edges, together with changes in band curvature, plays a decisive role in determining the transport coefficients. It is reported that Bads with higher curvature near the conduction or valence band edges correspond to lower carrier effective mass and higher mobility, thereby enhancing electrical conductivity.^54,58,59^ Conversely, flatter bands yield higher effective mass and reduced mobility, even when the DOS is large. Therefore, the combined effect of DOS magnitude and band-edge curvature will govern the observed trends in Seebeck coefficients, electrical conductivity and power factor under strain.

A potential difference (Δ*V*) is created between two junctions of distinct electrical conductors or semiconductors when a temperature gradient (Δ*T*) is placed between them, and this phenomenon is well known as the Seebeck effect.^12^ The physics underlying this can be explained using the concepts presented in the Fermi Dirac distribution function: a large percentage of the electron distribution at the hot junction occurs above the Fermi level, whereas it is negligible at the cold junction; the hot region's charge carriers acquire kinetic energy, making them more nimble than those at the cold region; as a result, carriers (either electrons above the Fermi level or holes below the Fermi level) diffuse from the high temperature to low temperature junction, which causes a difference in the chemical potential between the two junctions as a result of the shifting of the Fermi levels. This intriguing thermoelectric property is measured by the voltage difference (Δ*V*) produced per unit change in temperature (Δ*T*). This measurement is known as the Seebeck coefficient, represented as *S* = Δ*V*/Δ*T*. The calculation of transport parameters, as described in eqn (2) and (3), can be carried out using the Boltzmann Transport Equation (BTE) framework.^60^ Using the *ab initio* method combined with semiclassical Boltzmann transport theory within the constant relaxation time approximation (CRTA), we have evaluated the normalized thermoelectric parameters, including the Seebeck coefficient (*S*), electrical conductivity per relaxation time (*σ*/*τ*), thermal conductivity (*K*/*τ*) and the normalized thermoelectric power factor (PF/*τ*).

**Fig. 8:**
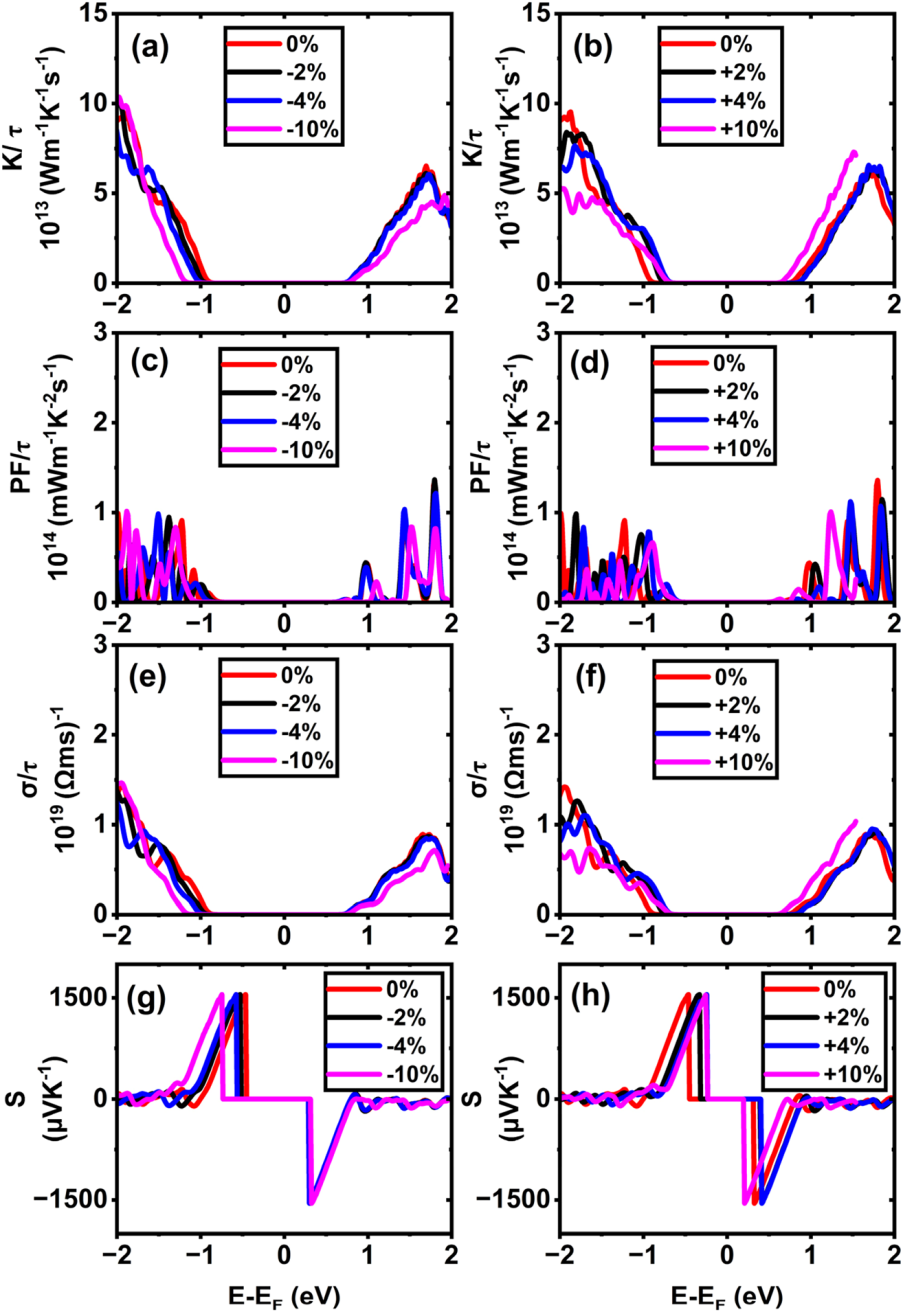
Calculated electronic transport properties of the (10,0) single-walled SiCNT as a function of chemical potential under different uniaxial strains: (a and b) thermal conductivity, (c and d) power factor, (e and f) electrical conductivity, and (g and h) Seebeck coefficient.

**Fig. 9:**
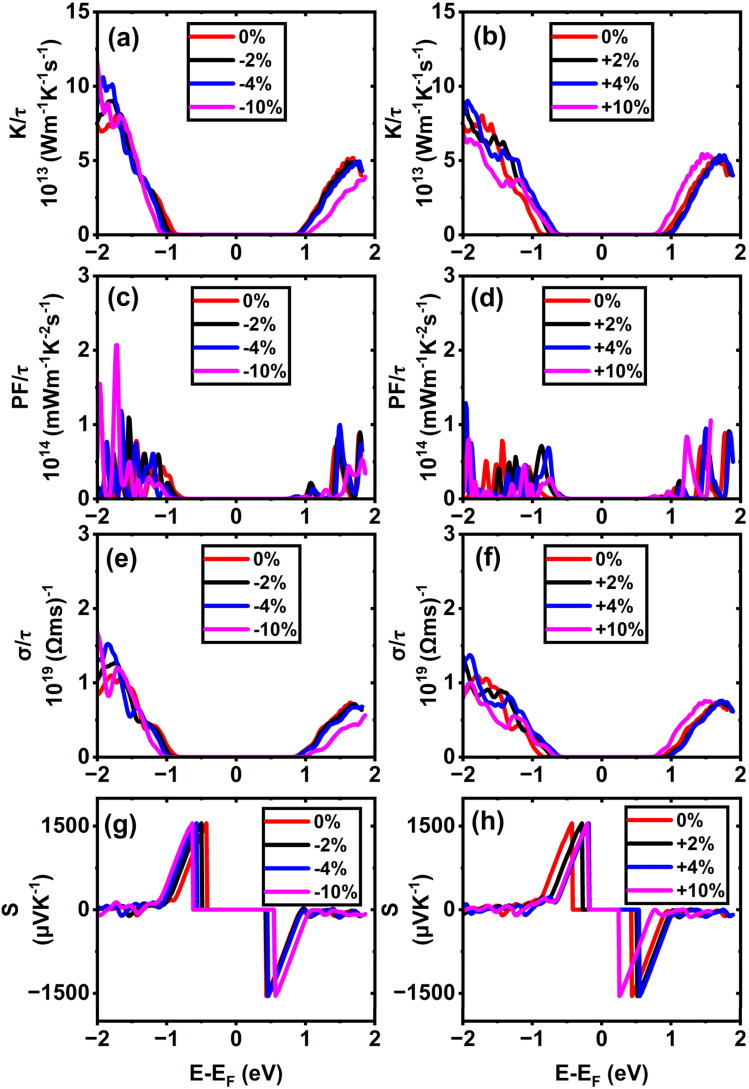
Calculated electronic transport properties of the (11,0) single-walled SiCNT as a function of chemical potential under different uniaxial strains: (a and b) thermal conductivity, (c and d) power factor, (e and f) electrical conductivity, and (g and h) Seebeck coefficient.

**Fig. 10:**
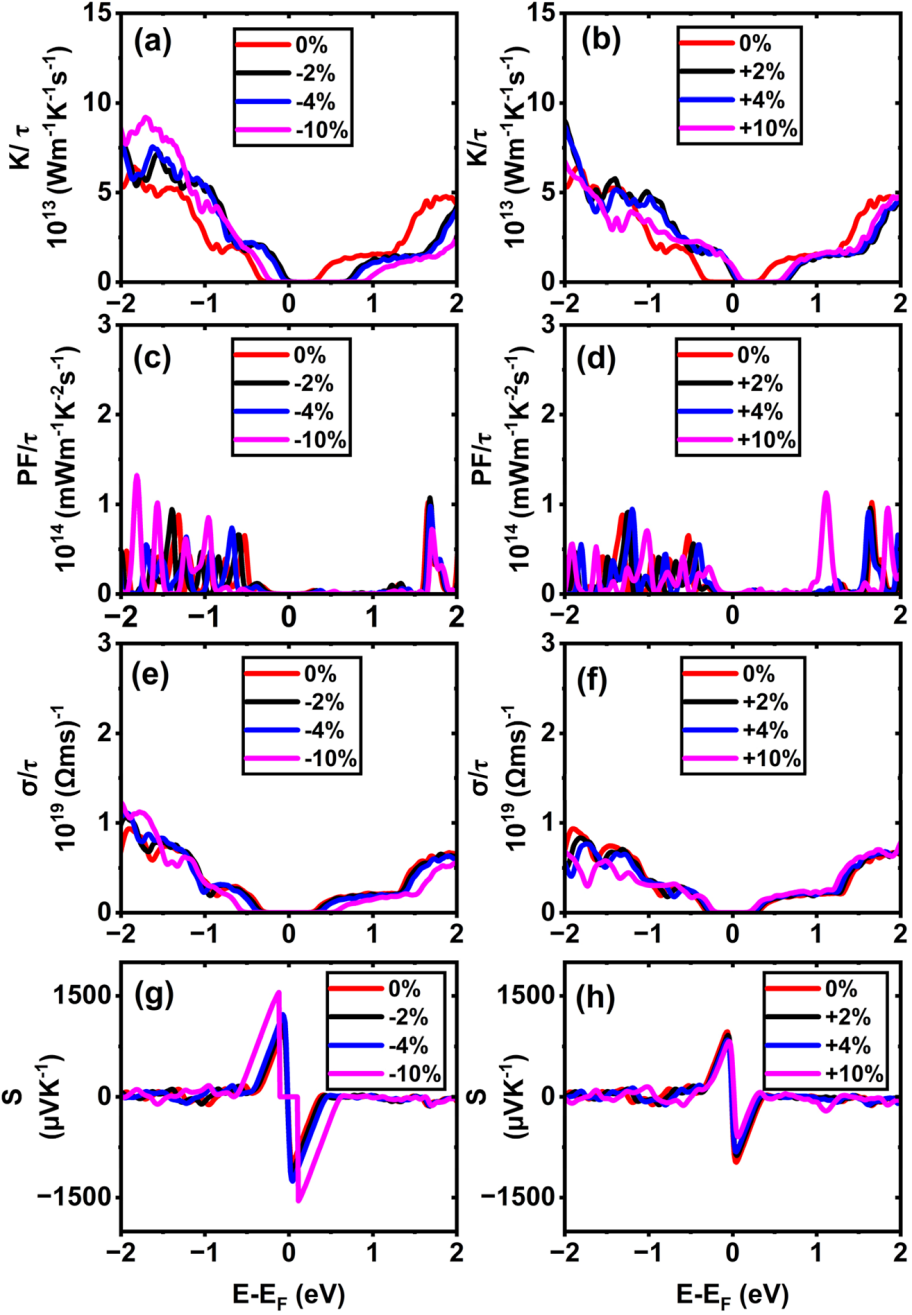
Calculated electronic transport properties of the (6,0) single-walled SiCNT as a function of chemical potential under different uniaxial strains: (a and b) thermal conductivity, (c and d) power factor, (e and f) electrical conductivity, and (g and h) Seebeck coefficient.

**Fig. 11:**
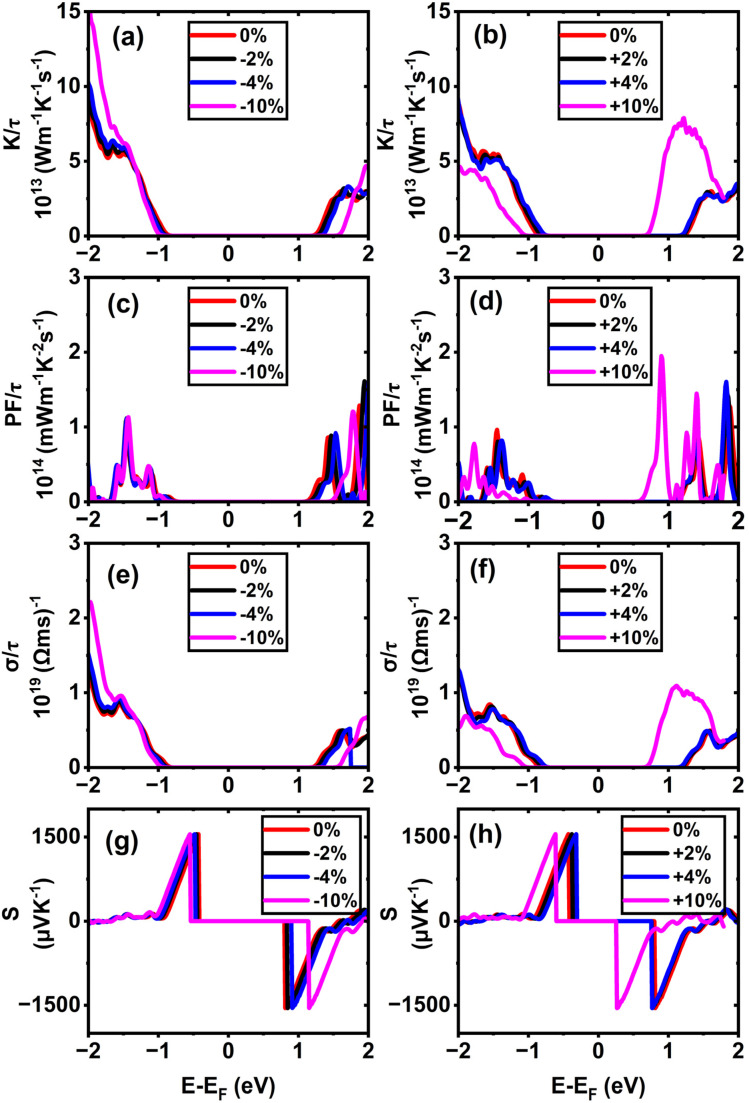
Calculated electronic transport properties of the (6,6) single-walled SiCNT as a function of chemical potential under different uniaxial strains: (a and b) thermal conductivity, (c and d) power factor, (e and f) electrical conductivity, and (g and h) Seebeck coefficient.

In (11,0) SWSiCNTs, the value of *S* is found to be 1550.24 µV K^−1^ at the unstrained condition, and it is almost constant for the application of strain −10% to +10% range (see Fig. 9). Such enhanced Seebeck coefficients near band edges at low carrier concentrations are well documented within the Boltzmann transport framework for low-dimensional semiconductors.^54,58^ Again, the value of *S* for (10,0) is found to be 1550.9 µV K^−1^ at the relaxed state (0% strain) and the +10% tensile strain takes the value for (10,0) to 1542.13 µV K^−1^ (see Fig. 8). Here, for the strain applied on these zigzag types along with the armchair (6,6) (see Fig. 11) shows a negligible variation of the Seebeck coefficient. Although compressive strain enhances the bandgap (see Fig. 6), the Seebeck coefficient remains almost constant at ∼1550 µV K^−1^. This stability can be attributed to the fact that strain does not significantly change the slope of the electronic transport distribution near the Fermi level. In addition, the Seebeck coefficient may already be close to saturation, where further bandgap widening has little effect. The strain-dependent band dispersion and DOS analysis further support this behavior. As evident from the band structures (see Fig. 2, 4 and S4), the curvature near the conduction and valence band edges remains nearly unchanged for (10,0), (11,0), and (6,6) SWSiCNTs over the considered strain range, implying minimal variation in the carrier effective mass. Consistently, the corresponding DOS near the Fermi level shows no abrupt redistribution under strain, and PDOS analysis confirms that the dominant Si-3p and C-2p orbital contributions near the band edges are preserved (see Fig. S8). Consequently, the energy derivative of the transport distribution function around the Fermi level remains nearly invariant, resulting in a strain-insensitive Seebeck coefficient. Such robustness is beneficial, as it allows tuning of electrical conductivity through strain while maintaining a high thermopower, thereby favoring an improved power factor. In contrast, (6,0) SWSiCNT shows a narrow bandgap of 0.67 eV, and the value of *S* is 965.05 µV K^−1^ at its relaxed state (see Fig. 10). The Seebeck coefficient (*S*) increases following a similar trend as the bandgap (*E*_g_) under compressive strain. *S* reached its maximum value of 1550.46 µV K^−1^ at 10% compressive strain. So, the variation of the Seebeck coefficient in the case of zigzag SWSiCNTs is not proportional to the bandgap except (6,0) SWSiCNT (see Fig. 6). SiCNTs with a direct bandgap and high Seebeck coefficient are ideal for thermoelectric devices in optoelectronic systems, where simultaneous light interaction and efficient heat-to-electricity conversion are desirable. These can be integrated into infrared sensors, photodetectors, or on-chip energy harvesters in light-exposed environments, offering multifunctionality in nano-optoelectronic and wearable technologies. Moreover, indirect bandgap SiCNTs with enhanced Seebeck coefficient performance are more suited for conventional thermoelectric generators, especially in high-temperature electronics and power systems. Their structural stability, combined with efficient thermoelectric behavior, makes them promising for waste heat recovery in microelectronics or aerospace thermal management systems, where light emission is not required but robust thermal-to-electrical conversion is critical.

Alongside the Seebeck coefficients, we qualitatively examine the influence of axial strain on the thermoelectric properties, with the electrical transport coefficients evaluated in normalized form (*σ*/*τ*, PF/*τ*, *K*/*τ*) within the constant relaxation time approximation, thereby ensuring that the discussion focuses on relative trends rather than absolute magnitudes. According to their respective DOS and *σ* plots, electrical conductivity *σ* for semiconducting SWSiCNTs is insignificant inside the charge neutrality point (CNP) because there are no energy states. Away from the CNP, *σ* is strongly influenced not only by the availability of electronic states (DOS) but also by the curvature of the contributing bands, which determines the carrier effective mass and mobility. The sharper curvature of the CBM indicates a lower effective mass of electron m*, which leads to higher electron mobility, 
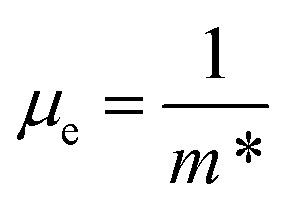
 , thereby enhancing electrical conductivity, *σ* = *qnµ*_e_. The effective mass is given by 
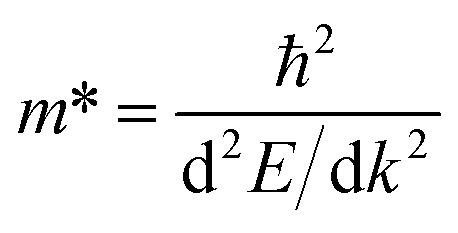
.^26^ However, due to application of tensile strain upon the (11,0) SWSiCNTs, the bandgap energy decreases (see Fig. S4) and the curvature of the CBM becomes sharp gradually, which leads to lower effective mass of the electron. When tensile strain is applied to the (11,0) SiCNT, it alters the atomic spacing and electronic interactions within the nanotube, which modifies its band structure. Specifically, the valence band (VB) becomes flatter under increasing tensile strain, indicating a reduction in the curvature near the valence band maximum. This flattening increases the effective mass of charge carriers, which directly leads to a decrease in carrier mobility in the n-type (11,0) SWSiCNTs. For (11,0) SWSiCNTs, *σ* decreases from 1.33 × 10^19^ (Ω ms)^−1^ to 8.08 × 10^18^ (Ω ms)^−1^due to the increment of tensile strain from +2% to +10% (see Fig. 9). For both the compressive and the tensile strain, there is a larger amount of conduction band (CB) energy states (see Fig. S5) compared to valence band (VB), which leads a higher *σ* at the left of 1st vHs. Likely (11,0), (10,0) shows a similar trend of *σ*/*τ* variation where due to tensile strain, 0% to +10%, the value of *σ*/*τ* decreases from 1.36 × 10^19^ (Ω ms)^−1^ to 6.81 × 10^18^ (Ω ms)^−1^ (see Fig. 8). For the maximum compressive strain, the highest number of energy states are found which causes highest *σ*/*τ* at n-type (10,0) SWSiCNT. It is noticeable that for both (10,0) and (11,0), the DOS remains almost fixed up to ±4% strain but it varies significantly for the ±10% strain in the right of 1st vHs which varies the conductivity respectively (see Fig. 8 and 9). In contrast, the (6,0) SWSiCNT exhibits an asymmetric response to axial strain (see Fig. 10). Under compressive strain, *σ*/*τ* increases due to the enhancement of conduction band states and partial suppression of curvature-induced band flattening, which improves carrier mobility. However, under tensile strain, *σ*/*τ* decreases despite the presence of available electronic states. This reduction is attributed to pronounced band flattening in this small-diameter nanotube, which increases the carrier effective mass and enhances scattering, thereby reducing mobility. This behavior highlights the strong interplay between curvature, strain, and transport in narrow SWSiCNTs and reflects the trade-off between carrier concentration and mobility commonly observed in strained low-dimensional systems.^61^ Though the zigzag type SWSiCNTs do not show the uniformity comparing to the bandgap but the armchair (6,6) shows the proportionate relationship. Here, the relaxed conductivity is 1.29 × 10^19^ (Ω ms)^−1^, and it increases to 2.25 × 10^19^ (Ω ms)^−1^ for the variation of compressive strain up to −10%. The opposite phenomena occur for the increment of tensile strain up to +10% which is exactly proportional to the bandgap energy variation of (6,6) SWSiCNTs (see Fig. 11). A very small amount of energy states is found for p-type (6,6) SWSiCNTs in case of maximum compressive strain, which ultimately lessened the *σ*/*τ*. Other than maximum strain almost constant *σ*/τ is found because of the same curvature of CBM and VBM, and also a constant number of energy states in the DOS (see Fig. 5).

The thermoelectric power factor, PF = *S*^2^*σ*, is one of the most important variables to evaluate a nanogenerator's TE performance. It is represented by the combined effect of *S* and *σ*. Despite the fact that *S*'s peak lies in the center of CNP, the PF/*τ* is minuscule there because there are no electronic energy states and, as a result, no electrical conductivity. Though there is a finite number of DOS, the PF/*τ* increases as *S* increases outside the first vHs. The PF/*τ* maxima occurs even though S moves very little at the outer region of CNP because the PF fluctuates proportionately to the square of *S*. In (11,0), the maximum value of PF/*τ* is found to be 2.07 × 10^14^ mW m^−1^ K^−2^ s^−1^ for maximum compressive strain, at 1.72 eV below the Fermi level (see Fig. 9). The PF/*τ* decreases uniformly with the decrease in compressive strain. And for the tensile strain, nonuniform variation of PF/*τ* is observed, suggesting a maximum 1.29 × 10^14^ mW m^−1^ K^−2^ s^−1^ PF for +4% strain at 1.96 eV below the Fermi level (*E*_F_) (see Fig. 9). Without adding strain, the maximum PF/*τ* value for (10,0) SWSiCNT is attained 1.36 × 10^14^ mW m^−1^ K^−2^ s^−1^ at 1.79 eV above the Fermi level (*E*_F_) (see Fig. 8) and it exists up to 2% compressive strain. The enhancement of PF can be primarily attributed to the successive improvement in *σ*, while the *S* remains nearly unchanged. Since PF is proportional to *S*^2^, the conductivity enhancement directly drives the observed increase in PF. Another semiconducting (6,0) SWSiCNT shows a maximum PF/*τ* value of 1.33 × 10^14^ mW m^−1^ K^−2^ s^−1^ at 1.81 eV below the Fermi level (*E*_F_) for 10% compressive strain (see Fig. 10). The value is almost 700 times the relaxed state at the same point. In the case of armchair (6,6) type SWSiCNTs, the maximum PF/*τ* is found for all uniaxial compressive strain at 1.43 eV below the Fermi level (*E*_F_) (see Fig. 11). And it is about 1.17 times the relaxed state at that point. In contrast, for tensile strain, decrement-natured PF/*τ* peak is observed.

In the context of energy-efficient thermal management systems, the electronic thermal conductivity, rather than the total phonon-dominated thermal conductivity, of nanomaterials like SWSiCNTs plays a crucial role and is the focus of the present analysis. Among the studied structures, the (10,0) zigzag SWSiCNT exhibits a maximum normalized electronic thermal conductivity of 9.53 × 10^13^ W m^−1^ K^−1^ s^−1^ at 1.87 eV below the Fermi level under relaxed state (see Fig. 8) and the value enhances to 10.4 × 10^13^ W m^−1^ K^−1^ s^−1^ at maximum compressive strain. This enhancement arises from strain-induced modifications of the electronic band structure, leading to increased carrier velocities and a higher contribution of conduction band states to heat transport. In contrast, tensile strain in (10,0) SWSiCNTs leads to a uniform reduction in normalized electronic thermal conductivity due to bandgap narrowing and changes in band curvature that reduce carrier mobility and energy transport efficiency. A similar trend is observed for the (11,0) SWSiCNT, where compressive strain leads to a monotonic increase in normalized electronic thermal conductivity, reflecting enhanced carrier transport associated with strain-modified electronic states near the Fermi level. The (6,0) SWSiCNT follows a similar overall trend to (10,0); its normalized electronic thermal conductivity reaches a maximum value of 9.20 × 10^13^ W m^−1^ K^−1^ s^−1^ at 10% compressive strain (see Fig. 10). Due to its smaller diameter and stronger curvature effects, strain induces more pronounced changes in band dispersion and DOS, resulting in a non-monotonic variation of normalized electronic thermal conductivity caused by competing effects of carrier concentration and effective mass. In contrast, the (6,6) armchair SWSiCNT responds differently; it shows a monotonic increase in normalized electronic thermal conductivity with compressive strain, reaching a maximum of >14.69 × 10^13^ W m^−1^ K^−1^ s^−1^ at −10% strain (see Fig. 11), and an opposite trend is observed for the tensile one. This behavior is attributed to the more symmetric electronic structure of armchair tubes, which leads to smoother strain-induced evolution of carrier velocities and electronic heat transport. Overall, zigzag SWSiCNTs exhibit greater strain sensitivity and a non-monotonic electronic thermal conductivity due to curvature-enhanced electronic-structure modulation, whereas armchair configurations show a more stable and predictable normalized electronic thermal transport response.

**Fig. 12:**
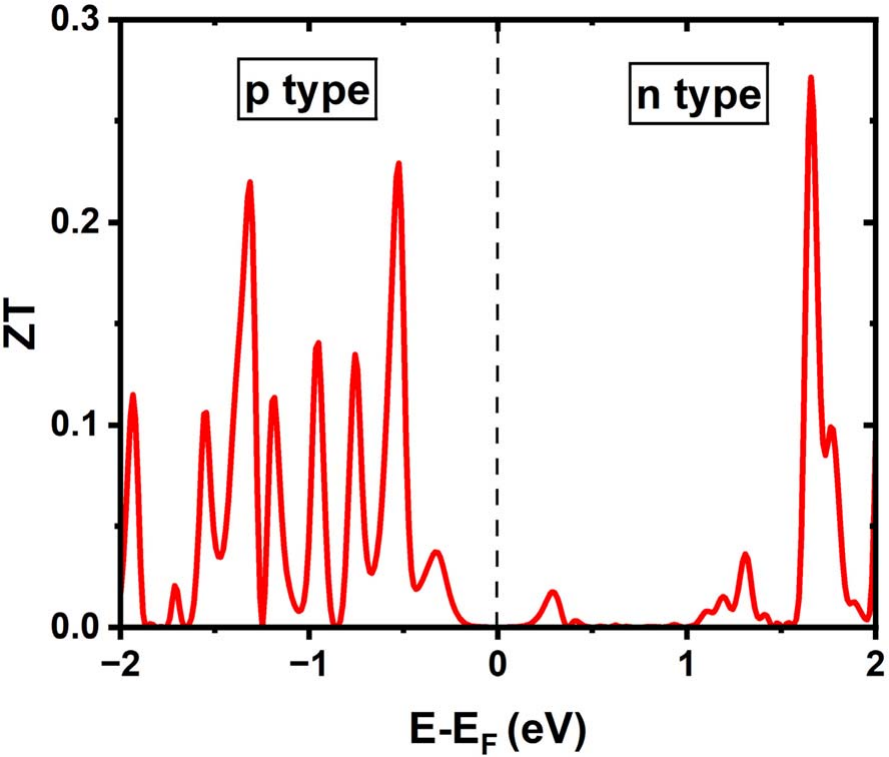
Variation of the thermoelectric figure of merit (*ZT*) with chemical potential *E* − *E*_F_ for pristine (6,0) SWSiCNT at 300 K. Negative and positive regions correspond to p-type and n-type doping, respectively.

To further evaluate the overall thermoelectric performance, the dimensionless thermoelectric figure of merit (*ZT*) is calculated for the pristine (6,0) SWSiCNT as a function of the chemical potential. In this analysis, the lattice thermal conductivity (*K*_*l*_) is calculated to be 68.815 W m^−1^ K^−1^ at 300 K and incorporated together with the electronic contribution to determine the total thermal conductivity. As shown in Fig. 12, the *ZT* profile exhibits several pronounced peaks away from the Fermi level, corresponding to the p-type and n-type doping regions. The maximum *ZT* reaches ∼0.27 in the n-type region (∼1.6–1.7 eV above *E* − *E*_F_ = 0), while comparatively smaller peaks appear in the p-type region, indicating excellent thermoelectric performance compared to CNT systems.^62,63^ Near the CNP, the *ZT* value approaches zero due to the absence of available electronic states and negligible electrical conductivity. The observed enhancement of *ZT* near the band edges originates from the combined effect of a high Seebeck coefficient and increased electrical conductivity associated with the one-dimensional electronic structure of the nanotube. These results suggest that pristine (6,0) SWSiCNTs can exhibit promising thermoelectric performance under suitable carrier doping conditions.

## 4. Conclusion

In conclusion, first-principles calculations coupled with Boltzmann transport theory are used to systematically investigate the effect of uniaxial strain on the electronic and thermoelectric properties of SWSiCNTs. Strain is found to be an effective tuning parameter for the bandgap: compressive strain consistently opened the gap, while tensile strain quenched it, with the variation traced to the movement of Dirac points and the redistribution of allowed k-lines. These bandgap trends, confirmed by the corresponding DOS, shows only a partial correlation with thermoelectric behavior, as the Seebeck coefficient remained remarkably stable around ∼1550 µV K^−1^ across both compressive and tensile strains for all investigated chiralities. This robustness highlights the potential of SWSiCNTs as reliable high-Seebeck thermoelectric materials. Electrical conductivity exhibits strong sensitivity to strain and chirality. In zigzag tubes such as (6,0), (10,0) and (11,0), tensile strain sharpens the CBM curvature and decrease effective mass, but also induce valence band flattening, ultimately lowering carrier mobility and reducing conductivity. Conversely, compressive strain enhances conductivity by increasing the density of conduction band states, especially near the first Van Hove singularity. The armchair (6,6) tube displays a more direct proportionality between conductivity and bandgap variation, with conductivity increasing under compressive strain and decreasing under tensile strain. The thermoelectric power factor (PF) revealed a delicate interplay between Seebeck stability and conductivity modulation. Maximal PF/*τ* values are observed under compressive strain, reaching 2.07 × 10^14^ mW m^−1^ K^−2^ s^−1^ in (11,0), 1.36 × 10^14^ mW m^−1^ K^−2^ s^−1^ in (10,0), and 1.33 × 10^14^ mW m^−1^ K^−2^ s^−1^ in (6,0) SWSiCNTs. In each case, the enhancement is driven primarily by conductivity gains, as the Seebeck coefficient remains nearly unchanged. Conversely, armchair (6,6) nanotube shows more modest PF/*τ* improvement, reflecting its more uniform electronic response to strain. Electronic thermal conductivity analysis further revealed the anisotropic nature of strain effects. Zigzag SWSiCNTs show pronounced strain sensitivity and even non-monotonic behavior due to phonon softening, enhanced scattering, and possible lattice instabilities. In contrast, the armchair (6,6) tube exhibits a monotonic increase in normalized electronic thermal conductivity under compressive strain (exceeding 14.69 × 10^13^ W m^−1^ K^−1^ s^−1^ at −10%) and a corresponding decrease under tensile strain, consistent with enhanced lattice packing and stiffened phonon modes. Furthermore, the calculated thermoelectric figure of merit (*ZT*) for the pristine (6,0) SWSiCNT reaches ∼0.27 at 300 K, highlighting its promising thermoelectric efficiency compared with other low-dimensional semiconductor nanotubes. However, the present study is based on idealized, defect-free structures, whereas intrinsic defects such as vacancies and antisites are unavoidable in realistic systems. Therefore, future investigations incorporating defect engineering could provide deeper insight into tuning the electronic and thermoelectric transport properties of SWSiCNTs. Similarly, doping—an effective strategy for optimizing carrier concentration and enhancing thermoelectric efficiency—has not been considered here and represents an important direction for future research. In addition, the formation of heterostructures and interfaces, such as SiCNT-CNT or SiCNT-2D material systems, which can significantly influence phonon scattering and band alignment, remains unexplored and offers promising opportunities for performance enhancement. It is also important to note that lattice thermal conductivity has been explicitly evaluated only for the (6,0) SWSiCNT in the calculation of the thermoelectric figure of merit (*ZT*). Extending such analysis to other chiralities could provide a more comprehensive understanding and may lead to further optimization strategies. From a methodological perspective, the use of the GGA-PBE functional may underestimate the bandgap; thus, employing more advanced approaches, such as hybrid functionals (*e.g.*, HSE06), would improve the quantitative accuracy of the predictions. Overall, this work establishes a fundamental understanding of chirality- and strain-dependent thermoelectric behavior in SWSiCNTs and provides a solid foundation for future studies aimed at designing high-performance, application-oriented thermoelectric nanodevices.

## Author contributions

Imam Hussain: investigation, methodology, software, data curation, writing – original draft. A. S. M. Jannatul Islam: conceptualization, investigation, validation, resources, project administration, funding acquisition, supervision, software, writing – original draft, writing – reviewing and editing. Tamanna Tanvin Tanha: investigation, data curation, writing – original draft. Md. Mafizul Islam: methodology, software, writing – reviewing and editing.

## Conflicts of interest

The authors declare no conflicts of interest.

## Supplementary Material

RA-016-D6RA00892E-s001

## Data Availability

The structures and data files used and/or analyzed in this study are provided in the supplementary information (SI). Supplementary information is available. See DOI: https://doi.org/10.1039/d6ra00892e.
