# Supplementary material for: Strain- and chirality-engineered tunability of electronic and thermoelectric properties in SiC nanotubes: insights from first-principles calculations
Source: RSC Adv. 2026 Apr 14;16(22):19570–87. doi: 10.1039/d6ra00892e (PMC13077544; doi:10.1039/d6ra00892e)
Supplement: RA-016-D6RA00892E-s001 [file RA-016-D6RA00892E-s001.pdf]

Supplementary Information (SI) for RSC Advances.
This journal is © The Royal Society of Chemistry 2026

Supplementary Information

Strain- and chirality-engineered tunability of electronic and thermoelectric properties in SiC nanotubes: insights from first-principles calculations

Imam Hussain[a], A. S. M. Jannatul Islam[a,*], Tamanna Tanvin Tanha[a], and Md. Mafizul Islam[b]

[a]Department of Electrical and Electronic Engineering, Khulna University of Engineering & Technology, Khulna 9203, Bangladesh.

[b]Department of Textile Machinery Design and Maintenance, Bangladesh University of Textiles, Dhaka 1208, Bangladesh

***Corresponding author. E-mail:** jannatul@eee.kuet.ac.bd

Optimized crystal structure of (10,0) SiCNT (CIF format)

```
#======================================================================
# CRYSTAL DATA
#----------------------------------------------------------------------
data_VESTA_phase_1

_chemical_name_common                  'nanotube'
_cell_length_a                         13.246300
_cell_length_b                         13.246300
_cell_length_c                         5.375500
_cell_angle_alpha                      90.000000
_cell_angle_beta                       90.000000
_cell_angle_gamma                      120.000000
_cell_volume                           816.843149
_space_group_name_H-M_alt              'P 1'
_space_group_IT_number                 1

loop_
_space_group_symop_operation_xyz
   'x, y, z'

loop_
   _atom_site_label
   _atom_site_occupancy
   _atom_site_fract_x
   _atom_site_fract_y
   _atom_site_fract_z
   _atom_site_adp_type
   _atom_site_U_iso_or_equiv
   _atom_site_type_symbol
```

C	1.0	0.873700	0.500000	0.333000	Uiso	? C
Si	1.0	0.873700	0.500000	0.000000	Uiso	? Si
C	1.0	0.922000	0.633300	0.833000	Uiso	? C
Si	1.0	0.922000	0.633300	0.500000	Uiso	? Si
C	1.0	0.929100	0.753600	0.333000	Uiso	? C
Si	1.0	0.929100	0.753600	0.000000	Uiso	? Si
C	1.0	0.894200	0.849100	0.833000	Uiso	? C
Si	1.0	0.894200	0.849100	0.500000	Uiso	? Si
C	1.0	0.820600	0.910400	0.333000	Uiso	? C
Si	1.0	0.820600	0.910400	0.000000	Uiso	? Si
C	1.0	0.715700	0.931500	0.833000	Uiso	? C
Si	1.0	0.715700	0.931500	0.500000	Uiso	? Si
C	1.0	0.589700	0.910400	0.333000	Uiso	? C
Si	1.0	0.589700	0.910400	0.000000	Uiso	? Si
C	1.0	0.454900	0.849100	0.833000	Uiso	? C
Si	1.0	0.454900	0.849100	0.500000	Uiso	? Si
C	1.0	0.324500	0.753600	0.333000	Uiso	? C
Si	1.0	0.324500	0.753600	0.000000	Uiso	? Si
C	1.0	0.211300	0.633300	0.833000	Uiso	? C
Si	1.0	0.211300	0.633300	0.500000	Uiso	? Si
C	1.0	0.126300	0.500000	0.333000	Uiso	? C
Si	1.0	0.126300	0.500000	0.000000	Uiso	? Si
C	1.0	0.078000	0.366700	0.833000	Uiso	? C
Si	1.0	0.078000	0.366700	0.500000	Uiso	? Si
C	1.0	0.070900	0.246400	0.333000	Uiso	? C
Si	1.0	0.070900	0.246400	0.000000	Uiso	? Si
C	1.0	0.105800	0.150900	0.833000	Uiso	? C
Si	1.0	0.105800	0.150900	0.500000	Uiso	? Si
C	1.0	0.179400	0.089600	0.333000	Uiso	? C
Si	1.0	0.179400	0.089600	0.000000	Uiso	? Si
C	1.0	0.284300	0.068500	0.833000	Uiso	? C

Si	1.0	0.284300	0.068500	0.500000	Uiso	?	Si
C	1.0	0.410300	0.089600	0.333000	Uiso	?	C
Si	1.0	0.410300	0.089600	0.000000	Uiso	?	Si
C	1.0	0.545100	0.150900	0.833000	Uiso	?	C
Si	1.0	0.545100	0.150900	0.500000	Uiso	?	Si
C	1.0	0.675500	0.246400	0.333000	Uiso	?	C
Si	1.0	0.675500	0.246400	0.000000	Uiso	?	Si
C	1.0	0.788700	0.366700	0.833000	Uiso	?	C
Si	1.0	0.788700	0.366700	0.500000	Uiso	?	Si

Optimized crystal structure of (11,0) SiCNT (CIF format)

```
#======================================================================
# CRYSTAL DATA
#----------------------------------------------------------------------
data_VESTA_phase_1

_chemical_name_common                  'nanotube'
_cell_length_a                         14.230400
_cell_length_b                         14.230400
_cell_length_c                         5.374600
_cell_angle_alpha                      90.000000
_cell_angle_beta                       90.000000
_cell_angle_gamma                      120.000000
_cell_volume                           942.564318
_space_group_name_H-M_alt              'P 1'
_space_group_IT_number                 1

loop_
```

```
_space_group_symop_operation_xyz
   'x, y, z'

loop_
   _atom_site_label
   _atom_site_occupancy
   _atom_site_fract_x
   _atom_site_fract_y
   _atom_site_fract_z
   _atom_site_adp_type
   _atom_site_U_iso_or_equiv
   _atom_site_type_symbol
   C    1.0   0.882400   0.500000   0.333100   Uiso  ? C
   Si   1.0   0.882400   0.500000   0.000000   Uiso  ? Si
   C    1.0   0.929100   0.624400   0.833100   Uiso  ? C
   Si   1.0   0.929100   0.624400   0.500000   Uiso  ? Si
   C    1.0   0.941100   0.738700   0.333100   Uiso  ? C
   Si   1.0   0.941100   0.738700   0.000000   Uiso  ? Si
   C    1.0   0.917300   0.833700   0.833100   Uiso  ? C
   Si   1.0   0.917300   0.833700   0.500000   Uiso  ? Si
   C    1.0   0.859700   0.901700   0.333100   Uiso  ? C
   Si   1.0   0.859700   0.901700   0.000000   Uiso  ? Si
   C    1.0   0.773000   0.937100   0.833100   Uiso  ? C
   Si   1.0   0.773000   0.937100   0.500000   Uiso  ? Si
   C    1.0   0.664100   0.937100   0.333100   Uiso  ? C
   Si   1.0   0.664100   0.937100   0.000000   Uiso  ? Si
   C    1.0   0.542000   0.901700   0.833100   Uiso  ? C
   Si   1.0   0.542000   0.901700   0.500000   Uiso  ? Si
   C    1.0   0.416400   0.833700   0.333100   Uiso  ? C
   Si   1.0   0.416400   0.833700   0.000000   Uiso  ? Si
   C    1.0   0.297700   0.738700   0.833100   Uiso  ? C
```

Si	1.0	0.297700	0.738700	0.500000	Uiso	? Si
C	1.0	0.195300	0.624400	0.333100	Uiso	? C
Si	1.0	0.195300	0.624400	0.000000	Uiso	? Si
C	1.0	0.117600	0.500000	0.833100	Uiso	? C
Si	1.0	0.117600	0.500000	0.500000	Uiso	? Si
C	1.0	0.070900	0.375600	0.333100	Uiso	? C
Si	1.0	0.070900	0.375600	0.000000	Uiso	? Si
C	1.0	0.058900	0.261300	0.833100	Uiso	? C
Si	1.0	0.058900	0.261300	0.500000	Uiso	? Si
C	1.0	0.082700	0.166300	0.333100	Uiso	? C
Si	1.0	0.082700	0.166300	0.000000	Uiso	? Si
C	1.0	0.140300	0.098300	0.833100	Uiso	? C
Si	1.0	0.140300	0.098300	0.500000	Uiso	? Si
C	1.0	0.227000	0.062900	0.333100	Uiso	? C
Si	1.0	0.227000	0.062900	0.000000	Uiso	? Si
C	1.0	0.335900	0.062900	0.833100	Uiso	? C
Si	1.0	0.335900	0.062900	0.500000	Uiso	? Si
C	1.0	0.458000	0.098300	0.333100	Uiso	? C
Si	1.0	0.458000	0.098300	0.000000	Uiso	? Si
C	1.0	0.583600	0.166300	0.833100	Uiso	? C
Si	1.0	0.583600	0.166300	0.500000	Uiso	? Si
C	1.0	0.702300	0.261300	0.333100	Uiso	? C
Si	1.0	0.702300	0.261300	0.000000	Uiso	? Si
C	1.0	0.804700	0.375600	0.833100	Uiso	? C
Si	1.0	0.804700	0.375600	0.500000	Uiso	? Si

Optimized crystal structure of (6,0) SiCNT (CIF format)

```
#======================================================================
# CRYSTAL DATA
#----------------------------------------------------------------------
data_VESTA_phase_1

_chemical_name_common                  'nanotube'
_cell_length_a                         9.319200
_cell_length_b                         9.319200
_cell_length_c                         5.385400
_cell_angle_alpha                      90.000000
_cell_angle_beta                       90.000000
_cell_angle_gamma                      120.000000
_cell_volume                           405.047361
_space_group_name_H-M_alt              'P 1'
_space_group_IT_number                 1

loop_
_space_group_symop_operation_xyz
   'x, y, z'

loop_
   _atom_site_label
   _atom_site_occupancy
   _atom_site_fract_x
   _atom_site_fract_y
   _atom_site_fract_z
   _atom_site_adp_type
   _atom_site_U_iso_or_equiv
   _atom_site_type_symbol
```

C	1.0	0.820400	0.500000	0.332400	Uiso	? C
Si	1.0	0.820400	0.500000	0.000000	Uiso	? Si
C	1.0	0.870000	0.685000	0.832400	Uiso	? C
Si	1.0	0.870000	0.685000	0.500000	Uiso	? Si
C	1.0	0.820400	0.820400	0.332400	Uiso	? C
Si	1.0	0.820400	0.820400	0.000000	Uiso	? Si
C	1.0	0.685000	0.870000	0.832400	Uiso	? C
Si	1.0	0.685000	0.870000	0.500000	Uiso	? Si
C	1.0	0.500000	0.820400	0.332400	Uiso	? C
Si	1.0	0.500000	0.820400	0.000000	Uiso	? Si
C	1.0	0.315000	0.685000	0.832400	Uiso	? C
Si	1.0	0.315000	0.685000	0.500000	Uiso	? Si
C	1.0	0.179600	0.500000	0.332400	Uiso	? C
Si	1.0	0.179600	0.500000	0.000000	Uiso	? Si
C	1.0	0.130000	0.315000	0.832400	Uiso	? C
Si	1.0	0.130000	0.315000	0.500000	Uiso	? Si
C	1.0	0.179600	0.179600	0.332400	Uiso	? C
Si	1.0	0.179600	0.179600	0.000000	Uiso	? Si
C	1.0	0.315000	0.130000	0.832400	Uiso	? C
Si	1.0	0.315000	0.130000	0.500000	Uiso	? Si
C	1.0	0.500000	0.179600	0.332400	Uiso	? C
Si	1.0	0.500000	0.179600	0.000000	Uiso	? Si
C	1.0	0.685000	0.315000	0.832400	Uiso	? C
Si	1.0	0.685000	0.315000	0.500000	Uiso	? Si

Optimized crystal structure of (6,6) SiCNT (CIF format)

```
#=======================================================================
# CRYSTAL DATA
#-----------------------------------------------------------------------
data_VESTA_phase_1
```

```
_chemical_name_common                  'nanotube'
_cell_length_a                         13.639000
_cell_length_b                         13.639000
_cell_length_c                         3.101300
_cell_angle_alpha                      90.000000
_cell_angle_beta                       90.000000
_cell_angle_gamma                      120.000000
_cell_volume                           499.619598
_space_group_name_H-M_alt              'P 1'
_space_group_IT_number                 1

loop_
_space_group_symop_operation_xyz
   'x, y, z'

loop_
   _atom_site_label
   _atom_site_occupancy
   _atom_site_fract_x
   _atom_site_fract_y
   _atom_site_fract_z
   _atom_site_adp_type
   _atom_site_U_iso_or_equiv
   _atom_site_type_symbol
   Si       1.0     0.877300     0.500000     0.000000    Uiso  ? Si
   C        1.0     0.929100     0.649200     0.000000    Uiso  ? C
   Si       1.0     0.935700     0.717800     0.500000    Uiso  ? Si
   C        1.0     0.909300     0.833900     0.500000    Uiso  ? C
   Si       1.0     0.877300     0.877300     0.000000    Uiso  ? Si
   C        1.0     0.779900     0.929100     0.000000    Uiso  ? C
```

Si	1.0	0.717800	0.935700	0.500000	Uiso	? Si
C	1.0	0.575400	0.909300	0.500000	Uiso	? C
Si	1.0	0.500000	0.877300	0.000000	Uiso	? Si
C	1.0	0.350800	0.779900	0.000000	Uiso	? C
Si	1.0	0.282200	0.717800	0.500000	Uiso	? Si
C	1.0	0.166100	0.575400	0.500000	Uiso	? C
Si	1.0	0.122700	0.500000	0.000000	Uiso	? Si
C	1.0	0.070900	0.350800	0.000000	Uiso	? C
Si	1.0	0.064300	0.282200	0.500000	Uiso	? Si
C	1.0	0.090700	0.166100	0.500000	Uiso	? C
Si	1.0	0.122700	0.122700	0.000000	Uiso	? Si
C	1.0	0.220100	0.070900	0.000000	Uiso	? C
Si	1.0	0.282200	0.064300	0.500000	Uiso	? Si
C	1.0	0.424600	0.090700	0.500000	Uiso	? C
Si	1.0	0.500000	0.122700	0.000000	Uiso	? Si
C	1.0	0.649200	0.220100	0.000000	Uiso	? C
Si	1.0	0.717800	0.282200	0.500000	Uiso	? Si
C	1.0	0.833900	0.424600	0.500000	Uiso	? C

Figure S1

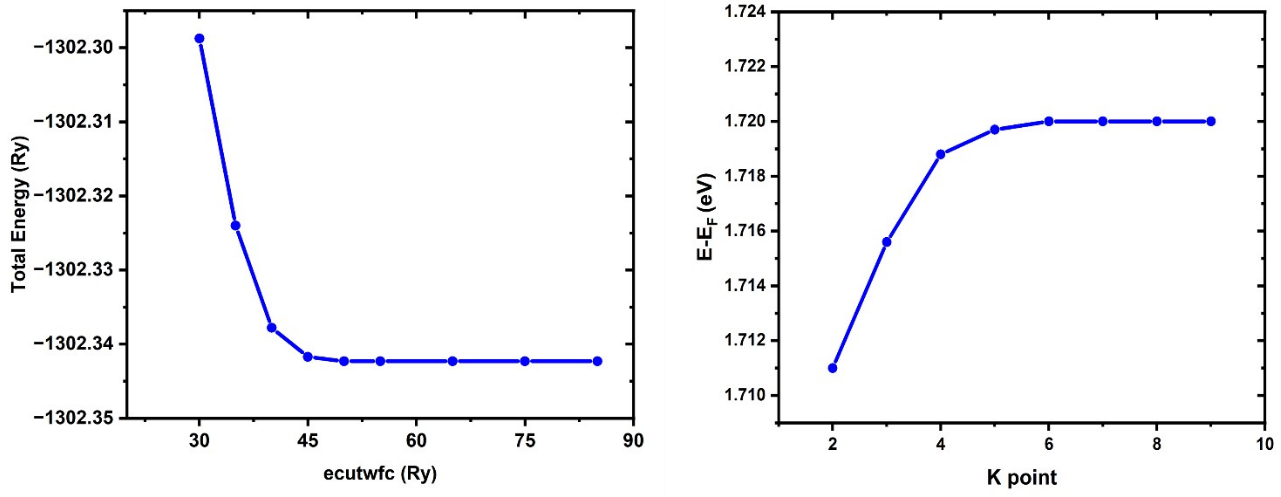

(a) Cuttoff energy convergence for (10,0) SiCNT

(b) k-point convergence of the bandgap for (10,0) SiCNT

Fig. S1. (a) Cutoff energy convergence for (10,0) SiCNT (b) k- point convergence of the bandgap for (10,0) SiCNT

Figure S2

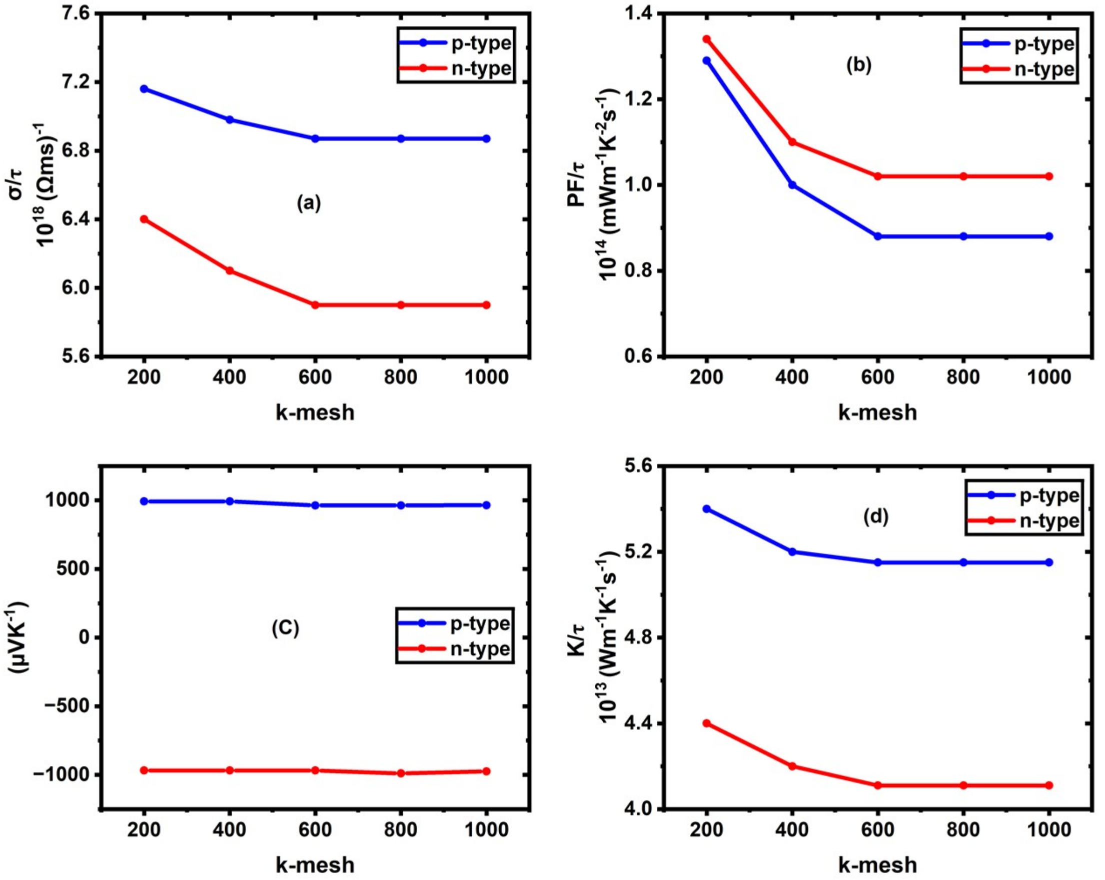

Fig. S2. k-mesh convergence of (a) electrical conductivity (σ/τ), (b) power factor (PF/τ), (c) Seebeck coefficient (S), and (d) electronic thermal conductivity (κ/τ) for the (6,0) SiCNT.

Figure S3

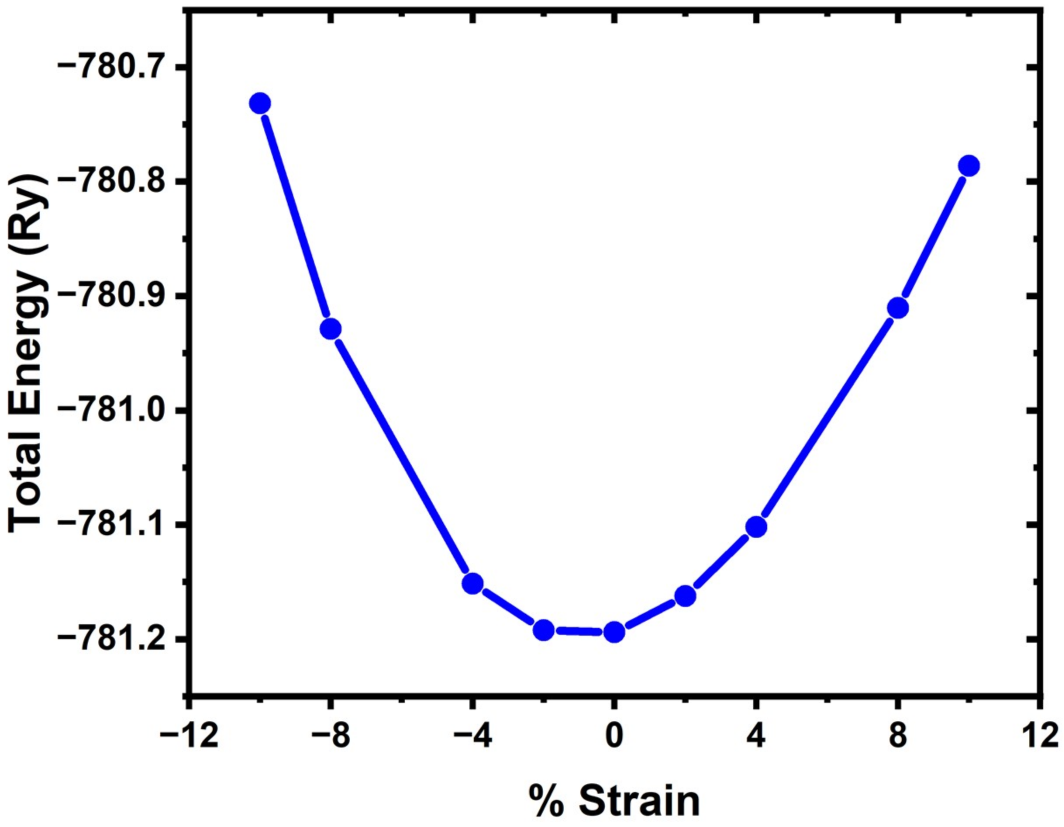

Fig. S3. Total energy as a function of applied uniaxial strain (−10% to +10%) for the (6,0) SiCNT

Figure S4

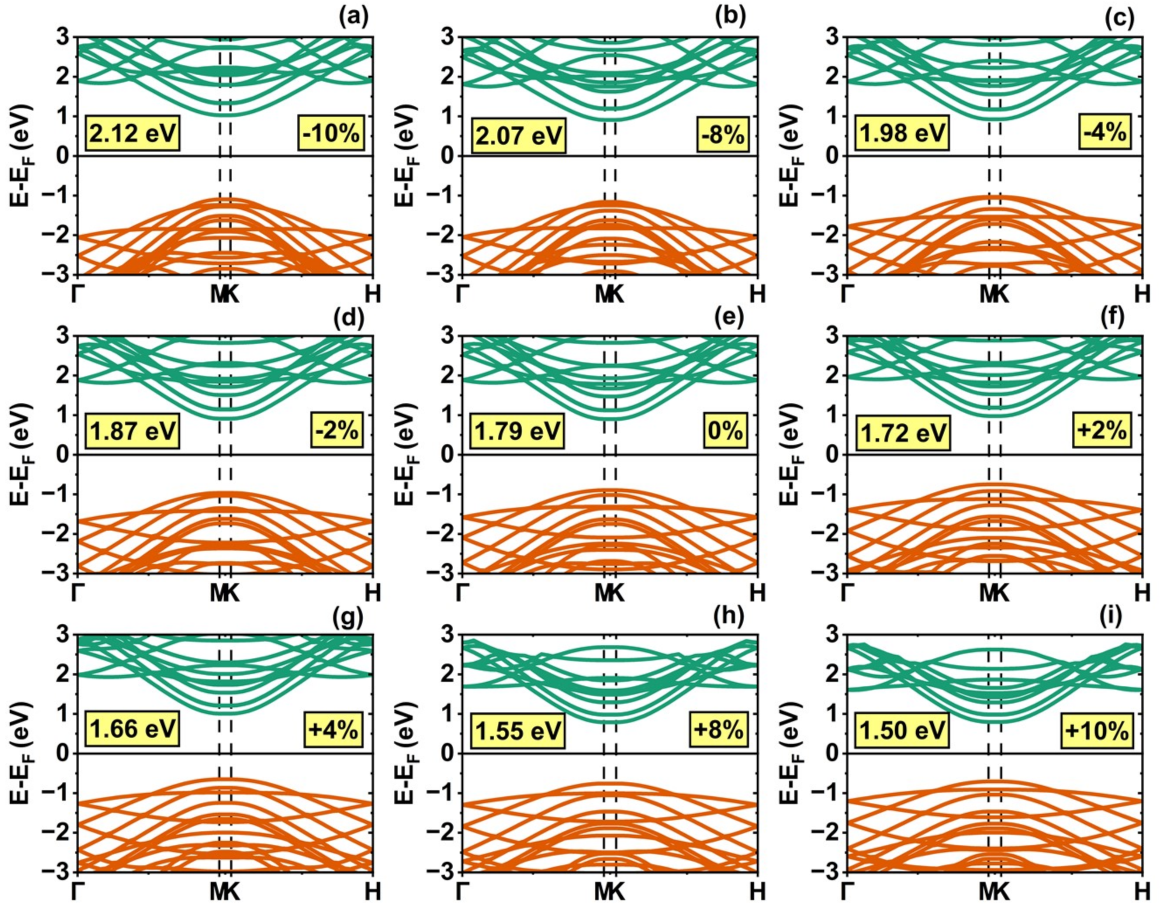

Fig. S4. Electronic band structures of the (11,0) single-walled SiCNT under uniaxial strain: (a) –10%, (b) –8%, (c) –4%, (d) –2%, (e) 0% (relaxed state), (f) +2%, (g) +4%, (h) +8%, and (i) +10%.

Figure S5

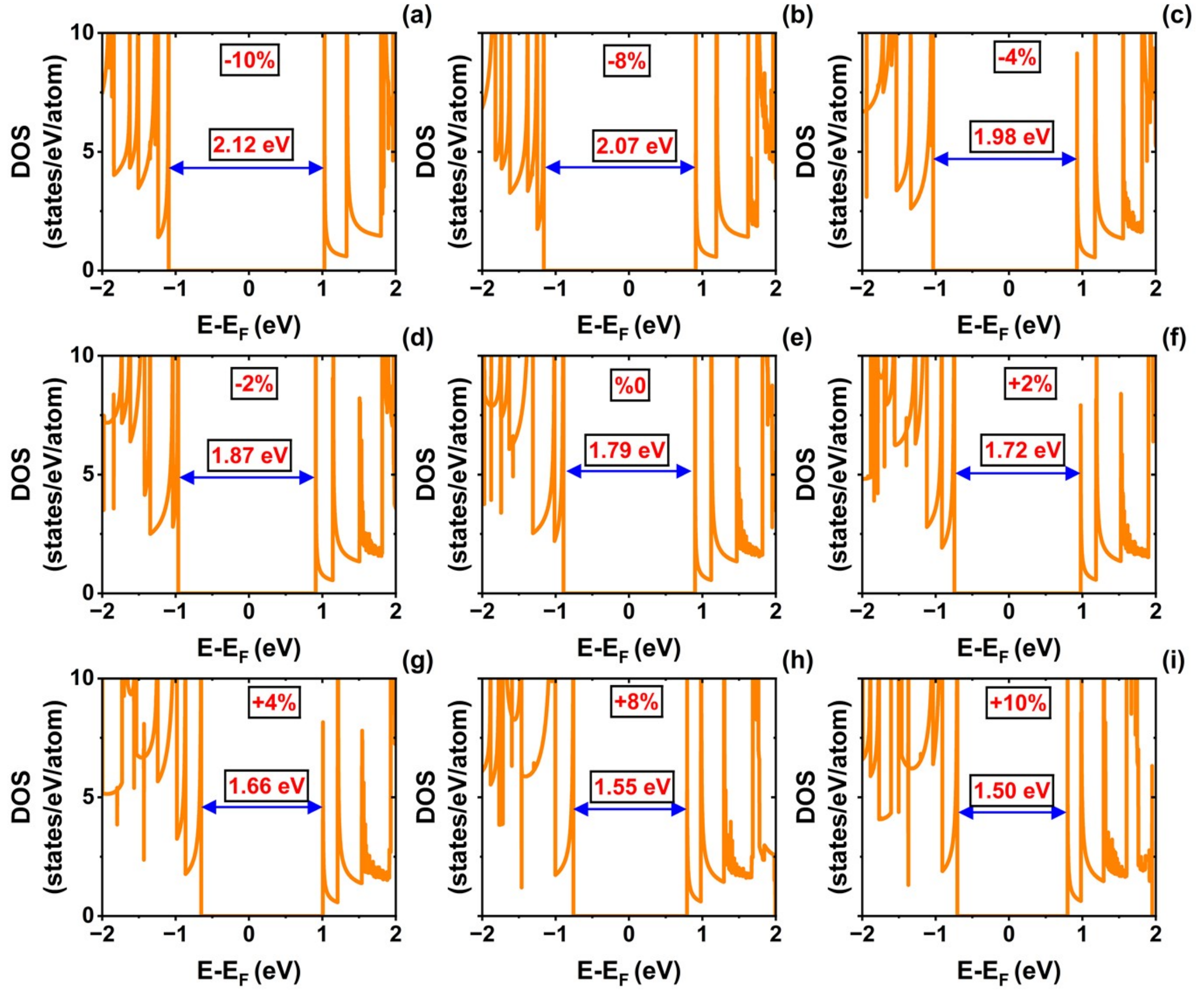

Fig. S5. Density of states (DOS) of the (11,0) single-walled SiCNT under uniaxial strain: (a) –10%, (b) –8%, (c) –4%, (d) –2%, (e) 0% (relaxed state), (f) +2%, (g) +4%, (h) +8%, and (i) +10%.

Figure S6

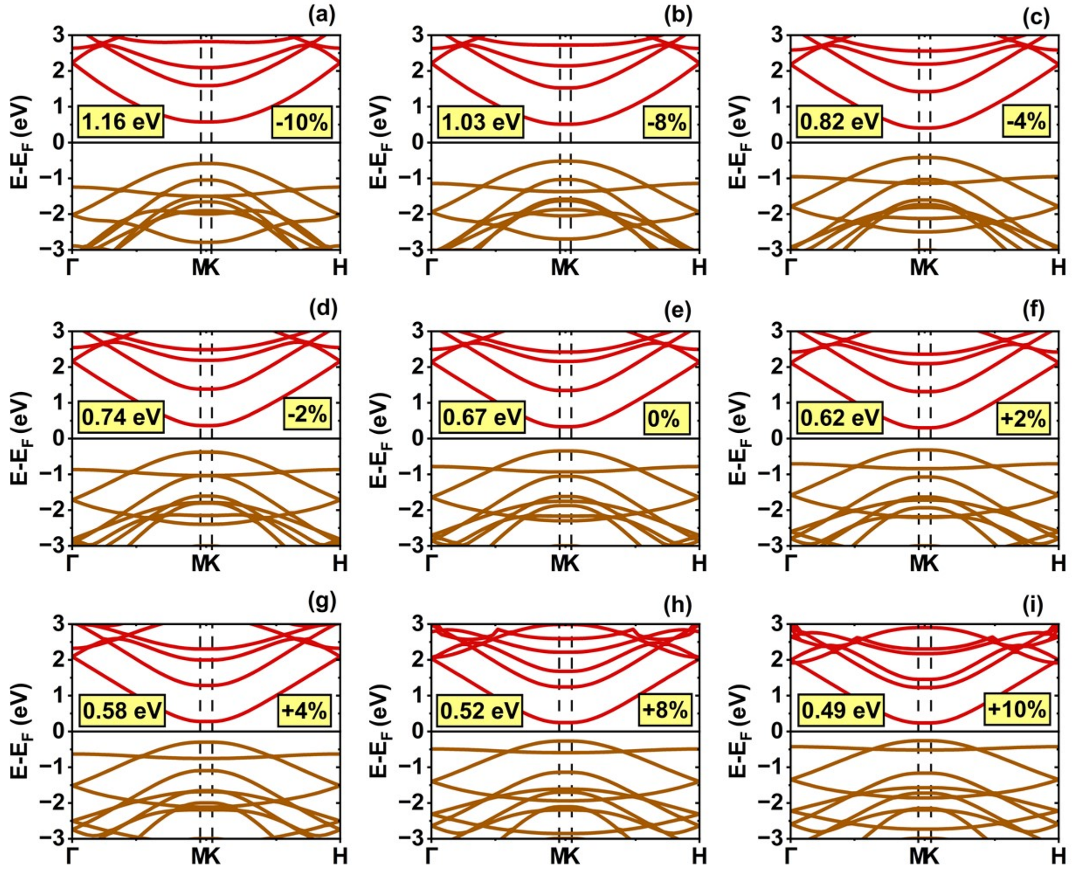

Fig. S6. Electronic band structures of the (6,0) single-walled SiCNT under uniaxial strain: (a) –10%, (b) –8%, (c) –4%, (d) –2%, (e) 0% (relaxed state), (f) +2%, (g) +4%, (h) +8%, and (i) +10%.

Figure S7

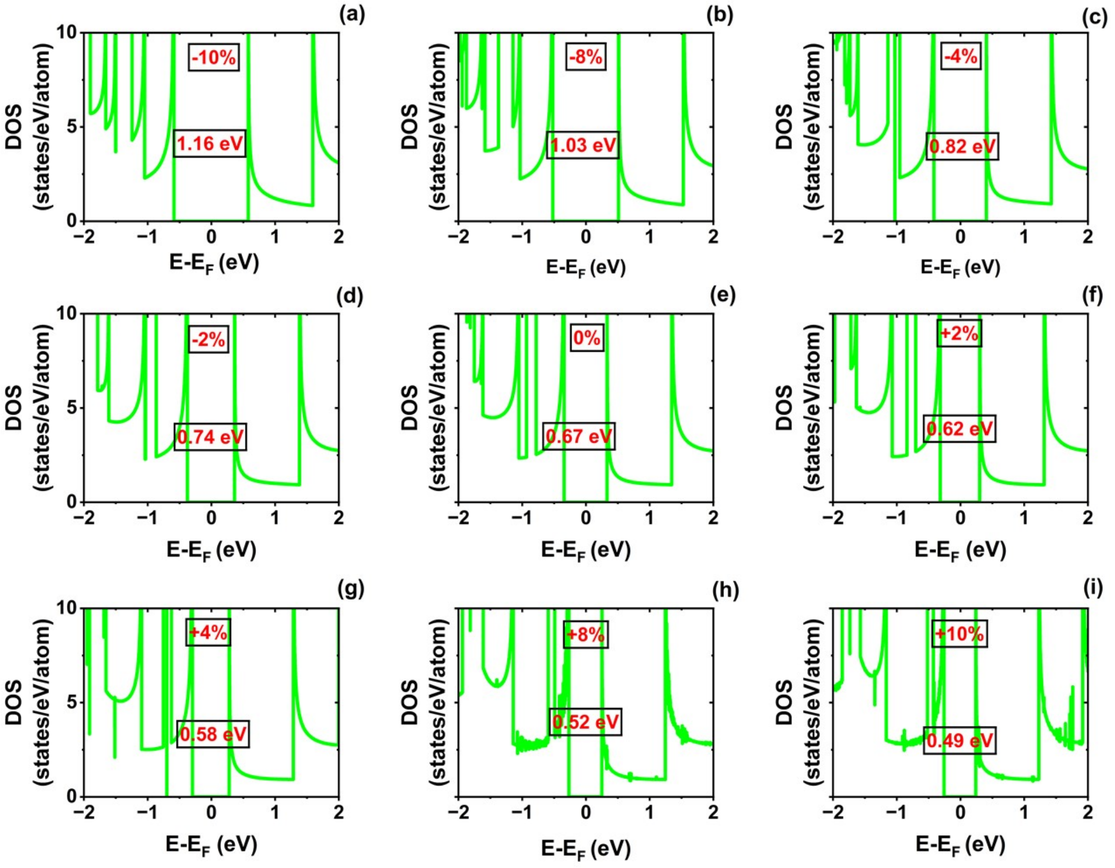

Fig. S7. Density of states (DOS) of the (6,0) single-walled SiCNT under uniaxial strain: (a) –10%, (b) –8%, (c) –4%, (d) –2%, (e) 0% (relaxed state), (f) +2%, (g) +4%, (h) +8%, and (i) +10%.

Figure S8

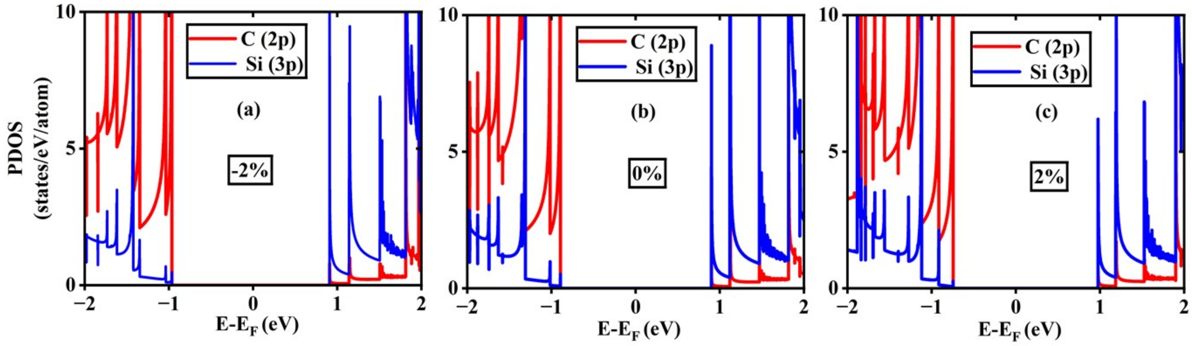

Fig. S8. Orbital-resolved PDOS of the (11,0) SiCNT under (a) −2%, (b) 0%, and (c) +2% strain, highlighting the contributions of C-2p and Si-3p states near the Fermi level.

Table S1. Comparison of bandgap values obtained using different exchange–correlation functionals and thermoelectric parameters of SiCNTs from literature and the present work

(A) Bandgap comparison using different theoretical methods for SWSiCNTs

Nanotube	GGA (present work)	GGA (Literature)	LDA	HSE06(estimated)[1]
(6,0)	0.67 eV	0.68 eV[2]	0.70 eV[3]	1.70 eV
(10,0)	1.72 eV	1.76 eV[2]	1.55 eV[4]	2.55 eV
(11,0)	1.79 eV	1.87 eV[2]	----	----
(6,6)	2.16 eV	2.03 eV[5]	1.78 eV[6]	2.78 eV

(B) Thermoelectric parameters from literature (SWCNTs)[7]

Nanotube	Seebeck(μVK^{-1})	σ/τ (Ω^{-1} m^{-1} s^{-1})	PF/τ (mW m^{-1} K^{-2} s^{-1})
(11,0)	1579 (-3%)	9.75×10^{18} (-3%)	1.55×10^{14} (-9%)
(10,0)	1580 (+3%)	9.5×10^{18} (-9%)	1.44×10^{14} (-3%)
(6,6)	155 (-6%)	2.03×10^{19} (-9%)	2.34×10^{14} (-9%)

(C) Thermoelectric parameters from the present work (SWSiCNTs)

Nanotube	Seebeck(μVK^{-1})	σ/τ (Ω^{-1} m^{-1} s^{-1})	PF/τ (mW m^{-1} K^{-2} s^{-1})
(11,0)	1550.24 (-10% to +10%)	1.72×10^{19} (-10%)	2.07×10^{14} (-10%)
(10,0)	1550.9 (0%)	1.46×10^{19} (-10%)	1.36×10^{14} (-0% to -2%)
(6,6)	1550 (-10% to +10%)	2.29×10^{19} (-10%)	1.95×10^{14} (+10%)

References

1 H. C. Hsueh, G. Y. Guo and S. G. Louie, Excitonic effects in the optical properties of SiC sheet and nanotubes, DOI:10.48550/ARXIV.1105.5531.

2 H. Wang, W. Liu and J. Zhao, Theoretical study on the oxidation of zigzag silicon carbide nanotubes (SiCNTs) by singlet O2, *Phys. B Condens. Matter*, 2012, **407**, 4238–4243.

3 I. J. Wu and G. Y. Guo, Optical properties of SiC nanotubes: An *ab initio* study, *Phys. Rev. B*, 2007, **76**, 035343.

4 R. J. Baierle, P. Piquini, L. P. Neves and R. H. Miwa, *Ab initio* study of native defects in SiC nanotubes, *Phys. Rev. B*, 2006, **74**, 155425.

5 W. Shi, S. Wu and Z. Wang, Tuning the electronic properties of single-walled SiC nanotubes by external electric field, *Phys. E Low-Dimens. Syst. Nanostructures*, 2016, **81**, 192–195.

6 Y. Qin, C. Chai, Z. Si, Y. Song and Y. Yang, Six novel silicon carbide with direct bandgaps: A comprehensive study, *Chem. Phys.*, 2022, **561**, 111603.

7 Md. M. Islam and A. Zubair, *Ab initio* study of uniaxial strain-induced thermoelectric property tuning of individual single-wall carbon nanotubes, *Mater. Adv.*, 2023, **4**, 6553–6567.
